# Voltage-gated calcium channels as key regulators of neuronal differentiation in the immortalized dorsal root ganglion neuronal cell line F11

**DOI:** 10.1038/s41598-026-44595-1

**Published:** 2026-03-23

**Authors:** D. López, J. Brea, M. Barro, M. I. Loza, A. L. Martínez

**Affiliations:** Innopharma Drug Screening and Pharmacogenomics Platform, BioFarma Research Group, Department of Pharmacology, Pharmacy and Pharmaceutical Technology, Center for Research in Molecular Medicine and Chronic Diseases (CiMUS), University of Santiago de Compostela and IDIS (Health Research Institute of Santiago de Compostela), Santiago de Compostela, Spain

**Keywords:** Cell biology, Neuroscience

## Abstract

Calcium signaling is essential for neuronal development and function, yet its role in the differentiation of immortalized neuronal cell lines remains poorly understood. In this study, we investigated the involvement of voltage-gated calcium channels (VGCCs) in the differentiation of the F11 cell line, a widely used translational model derived from the fusion of mouse neuroblastoma cells with rat dorsal root ganglion neurons. Differentiation was associated with greater KCl-evoked increases in intracellular Ca^2+^ and with upregulated expression of VGCC-encoding genes, particularly those for L-type channels. Pharmacological inhibition of L-type VGCCs reduced the KCl-evoked intracellular Ca^2+^ increase and impaired neurite outgrowth, underscoring their role in differentiation. Additionally, overexpression of Ca_V_1.3 in non-differentiated F11 cells enhanced neuronal features, including greater KCl-evoked increases in intracellular Ca^2+^ and neurite outgrowth. However, under differentiation conditions, Ca_V_1.3 overexpression disrupted the acquisition of neuronal features and coincided with increased intracellular oxidative stress. Notably, this effect was more pronounced for Ca_V_1.3 than for the higher activation-threshold Ca_V_1.2 channels. These findings highlight the dual role of L-type VGCCs in neuronal differentiation and oxidative stress regulation, demonstrating that their precise modulation is critical for proper neuronal development. Moreover, the shared molecular pathways between F11 cell differentiation and neurogenesis reinforce the translational value of this model for drug discovery efforts targeting oxidative stress-mediated neuronal dysfunction, such as in neurodegenerative diseases and neuropathic pain.

**Supplementary Information:**

The online version contains supplementary material available at 10.1038/s41598-026-44595-1.

## Introduction

Neuronal differentiation, a cornerstone of nervous system development, involves a finely tuned interplay of genetic and biochemical signals that shape key traits such as depolarization-evoked increases in intracellular Ca^2+^ and neurite outgrowth^1^. Elucidating the mechanisms underlying this process is critical for understanding and developing therapeutic strategies to treat nerve injuries and neurodegenerative disorders.

Calcium signaling is a fundamental regulator of various cellular functions, including neuronal development^2^. In neurons, voltage-gated calcium channels (VGCCs) mediate calcium influx in response to membrane depolarization, serving as key components of this signaling machinery^3^. This calcium entry plays a pivotal role in activating downstream pathways critical for neuronal differentiation^4^. One such pathway involves the calcium-dependent activation of the cAMP response element-binding (CREB) transcription factor, which is essential during the early stages of neuronal differentiation^2,3^. The calcium influx required for this activation triggers the MAPK/ERK (mitogen-activated protein kinase/extracellular signal-regulated kinase) and CaMK IV (calmodulin-dependent kinase IV) pathways, which ultimately phosphorylate and activate CREB^5^.

VGCCs are categorized based on their voltage dependence into high voltage-activated (HVA) and low voltage-activated (LVA) types^6^. The HVA category includes L-type, N-type, P/Q-type, and R-type channels, each characterized by unique functional and pharmacological properties^7^, while the LVA category consists only of T-type channels^8^.

In addition to their critical role in normal neuronal development, VGCCs are implicated in various pathophysiological processes^9^. Excessive calcium influx through VGCCs can disrupt cellular homeostasis, contributing to oxidative stress and neuronal damage in neurodegenerative diseases^10^. Furthermore, heightened calcium influx through VGCCs in dorsal root ganglion (DRG) neurons, which transmit pain signals from the limbs to the central nervous system, may lead to altered sensory perception and contribute to the pathogenesis of peripheral neuropathies and neuropathic pain^11^. Despite the clinical importance of VGCCs in these conditions, there is a scarcity of effective translational models that incorporate these channels for guiding drug discovery efforts targeting neuropathic pain.

Developing robust translational in vitro assays is essential for drug discovery, particularly in the field of brain therapeutics, where there remains a lack of new drugs to address unmet clinical need^12^. In this context, as there is a lack of new targets, phenotypic screening followed by target deconvolution is a key strategy. These models allow the identification of novel hits, followed by target deconvolution to pinpoint the specific molecular targets and cellular pathways responsible for the observed effects. This approach is crucial for uncovering the molecular mechanisms underlying therapeutic efficacy in early drug discovery^12^. Proper characterization of relevant signaling pathways is key to ensuring the translational applicability of these models.

In particular, the F11 cell line, an immortalized neuronal model derived from the fusion of mouse neuroblastoma and rat DRG neurons, has been widely used for studying neuropathic pain and screening novel analgesics^12–14^. Under standard culture conditions, F11 cells retain their proliferative capacity while maintaining the potential to differentiate and exhibit neuronal characteristics when treated with agents that elevate intracellular cAMP, such as forskolin, dibutyryl-cAMP, or IBMX (3-isobutyl-1-methylxanthine)^15^. This differentiation process leads to CREB activation, highlighting the importance of calcium and cAMP signaling in F11 cell differentiation^16^.

Our previous research demonstrated that sodium transients, mediated by voltage-gated sodium channels, contribute to F11 cell differentiation^17^. However, the role of calcium transients, specifically those mediated by VGCCs, remains insufficiently understood. Therefore, we hypothesize that calcium transients through VGCCs may play a major role in the regulation of F11 cell differentiation. Thus, this study aimed to: (i) characterize the involvement of VGCCs in F11 cell differentiation through genomic and pharmacological approaches, and (ii) elucidate the distinct contributions of Ca_V_1.2 and Ca_V_1.3 L-type VGCCs to the acquisition of neuronal phenotypic features, including the KCl-evoked increase in intracellular Ca^2+^ and neurite outgrowth, employing gene overexpression techniques. These findings will advance our understanding of the molecular mechanisms driving neuronal differentiation in this model and could provide insights into potential therapeutic strategies for promoting neuronal regeneration and function.

## Materials and methods

### Reagents

KCl (131494; Panreac-AppliChem, Castellar del Vallès, Barcelona, Spain) was freshly weighed and dissolved prior to each assay. Felodipine (F9677; Merck, Tres Cantos, Madrid, Spain), nitrendipine (N144; Merck), nifedipine (N7634; Merck) and Z944 (SML2635; Merck) were diluted in DMSO (dimethyl sulfoxide; D8418; Merck) at a stock concentration of 10 mM and stored at − 20 °C. ω-agatoxin (STA-500; Alomone Labs), ω-conotoxin (C-300; Alomone Labs), and SNX-482 (RTS-500; Alomone Labs) were diluted in ultrapure water at a stock concentration of 100 µM and stored at − 20 °C, following the manufacturer’s instructions. Aliquots were thawed only once and used shortly after preparation of working dilutions in differentiation medium containing 0.5% dialyzed FBS (foetal bovine serum; F0392; Merck).

### Cell culture and differentiation

Mouse neuroblastoma/rat embryonic DRG neuron hybrid F11 cells (08062601; ECCAC, Salisbury, England, UK) were cultured in Dulbecco’s modified Eagle’s medium (DMEM) without sodium pyruvate (D5671; Merck), supplemented with 10% (v/v) non-dialyzed FBS (F9665; Merck), 2 mM glutamine (G7513; Merck), 100 units/ml penicillin, and 100 µg/ml streptomycin (P0781; Merck) in a humidified atmosphere containing 5% carbon dioxide at 37 °C. F11 cells were routinely checked for mycoplasma contamination and tested negative.

Differentiation was achieved by exposing F11 cells in a humidified atmosphere with 5% carbon dioxide at 37 °C to differentiation medium containing 1 mM dibutyryl-cAMP (N6,2′-O-dibutyryladenosine 3′,5′-cyclic monophosphate; sc-201567; Santa Cruz Biotechnology, Heidelberg, Germany), 30 µM forskolin (sc-3562; Santa Cruz Biotechnology), 0.5% dialyzed FBS, 100 units/ml penicillin, 100 µg/ml streptomycin, and 2 mM glutamine in DMEM without sodium pyruvate for 72 h, as previously described^13^. Unless otherwise indicated, VGCC antagonists (including peptide toxins) were added at the start of the differentiation protocol and maintained throughout the 72 h period to assess long-term effects on the acquisition of neuronal features.

### Plasmid transfection

Both Ca_V_1.2 channel-encoding plasmid (Addgene plasmid #26572; http://n2t.net/addgene:26572; RRID: Addgene_26572) and Ca_V_1.3 channel-encoding plasmid (Addgene plasmid #49332; http://n2t.net/addgene:49332; RRID: Addgene_49332) were kind gifts from Diane Lipscombe^18,19^. The pcDNA3 empty vector was a kind gift from Marian Castro. All plasmids were purified using the Nucleobond Xtra Midi EF Kit (740420; Macherey-Nagel GmbH, Düren, Germany).

Forward transfection was carried out using a plasmid and FuGENE (E2311; Promega, Alcobendas, Madrid, Spain) mix in a 1:2 ratio. Cells were seeded in culture medium in the appropriate conditions for each experiment (see below). After 24 h, the medium was replaced with either low-FBS culture medium (0.5%) or differentiation medium, as required. The DNA and FuGENE mix solution in DMEM was then added to the wells. Transfection efficiency was verified by parallel transfection with a construct encoding GFP (S1 Fig), and overexpression was confirmed via RT-qPCR (S1 Table).

### Measurement of calcium transients

F11 cells were seeded into clear, flat-bottomed, black-walled, 384-well plates (781091; Greiner Bio-One, Frickenhausen, Baden-Württemberg, Germany) pretreated with 2 µg/ml laminin (L2020; Merck) and 20 µg/ml poly-D-lysine (P6407; Merck) at a density of 5000 cells/well in 50 µL of culture medium. After 24 h, medium was replaced in the corresponding wells with either differentiation medium or transfection reagents, as required. After 72 h, the medium was removed and replaced with 25 µL of fresh medium, then incubated with 25 µL of FLIPR Calcium-6 dye (R8190; Molecular Devices, Sunnyvale, CA) diluted in HBSS (Hank’s Balanced Salt Solution; 14065-049; Gibco, Invitrogen, Carlsbad, CA, USA) containing 20 mM HEPES [(4-(2-hydroxyethyl)-1-piperazineethanesulfonic acid); H3375; Merck] (pH 7.4) for 2 h at 37 °C.

Intracellular Ca²⁺ signals were recorded with an FDSS7000EX Functional Drug Screening System (Hamamatsu Photonics, Cerdanyola del Vallès, Barcelona, Spain) at 2 Hz (one frame every 0.5 s), exciting the calcium-sensitive dye at 470–495 nm and collecting emission at 515–575 nm. Excitation intensity and acquisition settings were kept constant across plates and conditions. FDSS dispensing tips (A8687-62; Hamamatsu Photonics) were pretreated with 0.1% BSA in assay buffer to minimize nonspecific adsorption. For each well, a baseline (F₀) was calculated as the mean fluorescence over frames 0–359 (first 180 s) prior to stimulation. At t = 180 s (frame 360), the instrument dispensed KCl (final concentration 30 mM). Raw traces F(t) were converted to baseline-normalized ratio traces R(t) = F(t)/F₀. The KCl-evoked Ca²⁺ signal amplitude was summarized as the peak-to-trough amplitude (max R–min R) within frames 360–1080 (180–540 s post-stimulus). Where indicated, amplitudes were normalized to the matched vehicle control on the same plate (set to 100%). FDSS recordings provide well-averaged fluorescence traces that quantify depolarization-evoked intracellular Ca²⁺ signals at the population level and do not directly measure action potential firing or membrane excitability.

Calcium levels during differentiation were measured in clear, flat-bottomed, black-walled, 96-well plates (6055308; Revvity, Tres Cantos, Madrid, Spain) pretreated with 30 µg/ml poly-d-lysine. Cells were seeded at a concentration of 7500 cells per well in 100 µL of culture medium. After 24 h, medium was replaced in half of the plates with 50 µL of differentiation medium, while the other half received 50 µL of fresh culture medium. FLIPR Calcium-6 dye (50 µL) was added to all wells, and the plates were analyzed using an Operetta CLS High-Content Analysis System (Revvity). Fluorescence readings were taken at 5, 10, 20, 30, 40, 60, and 70 h, using a 475 nm excitation/525 nm emission wavelength with a 5 × objective. Image analysis was conducted with Harmony High Content Imaging and Analysis Software (Revvity), considering mean fluorescence intensity inside the F11 cells, corresponding to intracellular Ca^2+^ concentration.

### Microscopy assays

Cells were seeded in 100 µL of culture medium at a density of 7,500 cells per well into clear-bottom, 96-well plates pretreated with 20 µg/ml poly-d-lysine. After 24 h, the culture medium was replaced in the corresponding wells with either differentiation medium or transfection reagents, as required. 72 h after medium replacement or transfection, F11 cells were fixed in 4% paraformaldehyde (sc-281692; Santa Cruz Biotechnology) in PBS (phosphate-buffered saline) solution at 4 °C for 20 min. The cells were washed twice with HBSS and permeabilized using blocking buffer containing 5% BSA and 0.1% Triton X-100 (T8787; Merck) in HBSS for 30 min at room temperature. Cells were then stained with Alexa 488 dye-conjugated anti-β-tubulin mouse antibody (558605; Becton, Dickinson and Company Biosciences, San Agustín de Guadalix, Madrid, Spain), diluted 1:500, and 2.5 µM nuclear stain DRAQ5 (108410; Abcam, Cambridge, UK) in DMEM for 1 h at room temperature. A High Content Imaging System Operetta CLS was used to acquire bright field images and fluorescence signals (20x WD objective, 25 fields per well), using a 475 nm excitation/525 nm emission wavelength for Alexa 488, and a 630 nm excitation/708 nm emission wavelength for DRAQ5. Image analysis was conducted using Harmony High Content Imaging and Analysis Software to measure the maximum neurite length in each well.

Ca_V_1.2 and Ca_V_1.3 VGCCs staining was performed in fixed F11 cells processed under both non-permeabilized (plasma membrane) and permeabilized (whole-cell) conditions. Cells were exposed to a solution containing anti-Ca_V_1.2 channel (ACC-003; Alomone Labs) and anti-Ca_V_1.3 channel rabbit antibodies (ACC-005; Alomone Labs), each diluted 1:500 in DMEM at 4 °C. Eighteen hours later, the cells were washed and incubated with a solution of goat anti-rabbit antibody conjugated with Alexa Fluor 594 (A11072; Thermo), diluted 1:1000 and 1 µg/ml Hoechst 33,342 (H3570; Merck) in HBSS for 1 h at room temperature. After washing twice with HBSS, a High Content Imaging System Operetta CLS was used to acquire bright-field images and fluorescence signals (20× WD objective, 36 fields per well), using a 365 nm excitation/465 nm emission wavelength for Hoechst, a 475 nm excitation/525 nm emission wavelength for Alexa 488, and a 545 nm excitation/610 nm emission wavelength for Alexa 594. Image analysis was conducted using Harmony High Content Imaging and Analysis Software by measuring the intensity of Alexa Fluor 594 fluorescence corresponding to channel protein expression either at the cell membrane in non-permeabilized cells or across the whole-cell area in permeabilized cells, as appropriate for each condition.

Intracellular ROS (reactive oxygen species) levels were assessed using CellROX Green reagent (C10444; Thermo Fisher, Alcobendas, Madrid, Spain), following the manufacturer’s protocol. Briefly, cells were incubated with 5 µM CellROX Green and 2.5 µM Hoechst 33,342 for 30 min at 37 °C. After incubation, cells were washed three times with PBS and imaged using the High Content Imaging System Operetta CLS. Fluorescence signals were captured with a 20x WD objective across 25 fields per well, using a 365 nm excitation/465 nm emission wavelength for Hoechst and a 475 nm excitation/525 nm emission wavelength for CellROX Green. The intensity of CellROX Green fluorescence in F11 cells was quantified using Harmony High Content Imaging and Analysis Software.

For all imaging-based assays, quantitative readouts were computed as well-level summaries derived from all segmented cells across the acquired fields of view, with each well considered one technical replicate.

### Real time reverse polymerase chain reaction (RT-qPCR)

RNA for RT-qPCR was isolated from confluent 6-well plates (353046; Thermo Fisher) using the RNeasy Mini Kit (Qiagen, Hilden, Germany). RT-qPCR was performed using 5 ng of RNA with the EXPRESS One-Step Superscript RT-qPCR kit (11781200; Thermo Fisher). Gene expression was measured using TaqMan assays on a QuantStudio 12 K Flex reader (Life Technologies, Carlsbad, CA, USA). Gene-specific probes and primers were obtained from Applied Biosystems (Foster City, CA, USA). The following TaqMan gene expression assays were used: *Cacna1a* (Mm00432190_m1), *Cacna1b* (Mm01333677_m1), *Cacna1c* (Mm01188822_m1), *Cacna1d* (Mm01209927_g1), *Cacna1e* (Mm00494444_m1), *Cacna1f* (Mm00490443_m1), *Cacna1g* (Mm00486572_m1), *Cacna1h* (Mm00445382_m1), *Cacna1i* (Mm01299033_m1), *Cacna1s* (Mm00489257_m1). All templates were analyzed in triplicate across three independent experiments, and the quantification cycle (C_q_) value of each gene was normalized to *36b4* gene using the formula ΔC_q_ = C_q_ (examined gene) - C_q_ (*36b4*). Gene expression comparisons between differentiated and non-differentiated cells were expressed as fold change (F.C.) values calculated using the ${2}^{-\varDelta\varDelta{C}_{q}}$ method.

### Data analysis

Data analysis was performed using GraphPad Prism 10.2 software (GraphPad Software, La Jolla, CA, USA). Concentration–response curves were fitted using a sigmoidal dose–response model to determine key parameters such as IC_50_ and maximum inhibition values (I_max_). Statistical comparisons of KCl-evoked intracellular Ca^2+^ signals, expression of VGCC-encoding genes, and channel protein expression between non-differentiated and differentiated F11 cells were conducted using Welch’s *t*-test. Differences in neurite length, intracellular Ca^2+^ levels, and ROS production between transfected and control cells were evaluated using one-way ANOVA, followed by Dunnett’s post-hoc test for multiple comparisons. Similarly, drug-induced changes in neurite length were analyzed using ANOVA with Dunnett’s post-hoc analysis to compare treatment groups to non-treated controls. All statistical tests were conducted under the assumption of normal data distribution and homogeneity of variances, verified where applicable. A p value < 0.05 was considered statistically significant for all analyses.

## Results

### Differentiation increases the KCl-evoked intracellular Ca^2+^ signal and upregulates VGCC-encoding gene expression

**Fig. 1:**
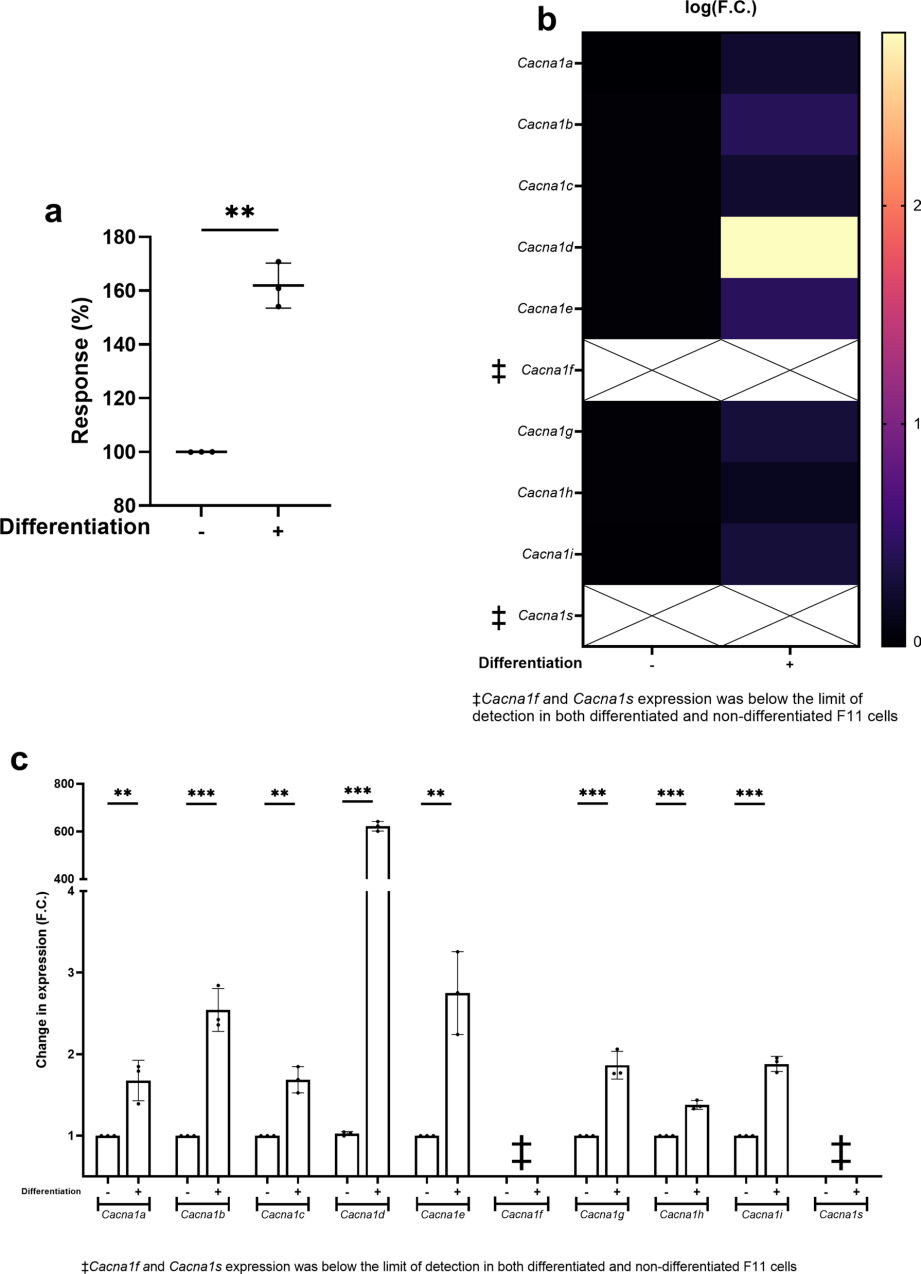
F11 cell differentiation increased the KCl-evoked intracellular Ca^2+^ signal and upregulated VGCC-encoding gene transcription. **a** Intracellular Ca²⁺ signals evoked by 30 mM KCl in non-differentiated (-) and differentiated F11 cells (+). Results are presented as mean ± S.D. from three independent experiments (*N* = 3), each with 3 technical replicates (*n* = 3). Values are expressed as a percentage relative to the response observed in non-differentiated F11 cells, which is set at 100%. **b** Heatmap representing the increase in the expression of genes encoding the α subunits of VGCCs in differentiated F11 cells (+) compared to non-differentiated F11 cells (-), expressed as mean logarithmic fold change [log(F.C.)] values. **c** Fold change (F.C.) increase (mean ± S.D.) in the expression of genes encoding the α subunits of VGCCs in differentiated F11 cells (+) compared to non-differentiated F11 cells (-). Results are presented as mean ± S.D. from three independent experiments (*N* = 3), each with 3 technical replicates (*n* = 3). ***p < 0.001, **p < 0.01; Welch’s *t* test.

Differentiation enabled the F11 cell line to respond to 30 mM KCl with a 62% higher intracellular Ca²⁺ signal than non-differentiated cells (*p* < 0.01) (Fig. 1a) (S2 Fig, panel a). In an orthogonal electrophysiological readout, differentiated F11 cells showed increased spontaneous spiking activity measured by multielectrode arrays (S3 Fig). This larger KCl-evoked intracellular Ca^2+^ signal was accompanied by a significant upregulation of genes encoding the α subunits of eight VGCCs (*Cacna1a*, *Cacna1b*, *Cacna1c*, *Cacna1d*, *Cacna1e*, *Cacna1g*, *Cacna1h*, and *Cacna1i*) (*p* < 0.01) (Fig. 1b, c) (S2 Table). Among these, *Cacna1d*, which encodes the L-type Ca_V_1.3 channel^20^, exhibited the highest level of upregulation, with a 620-fold increase in expression following differentiation. In contrast, as discussed below, *Cacna1f* and *Cacna1s* were not expressed in either non-differentiated or differentiated F11 cells, consistent with their expression being restricted to the retina and skeletal muscle^21,22^.

### Pharmacological blockade of Ca_V_1.2 and Ca_V_1.3 VGCCs impaired the acquisition of neuronal phenotypic features of F11 cells

**Fig. 2:**
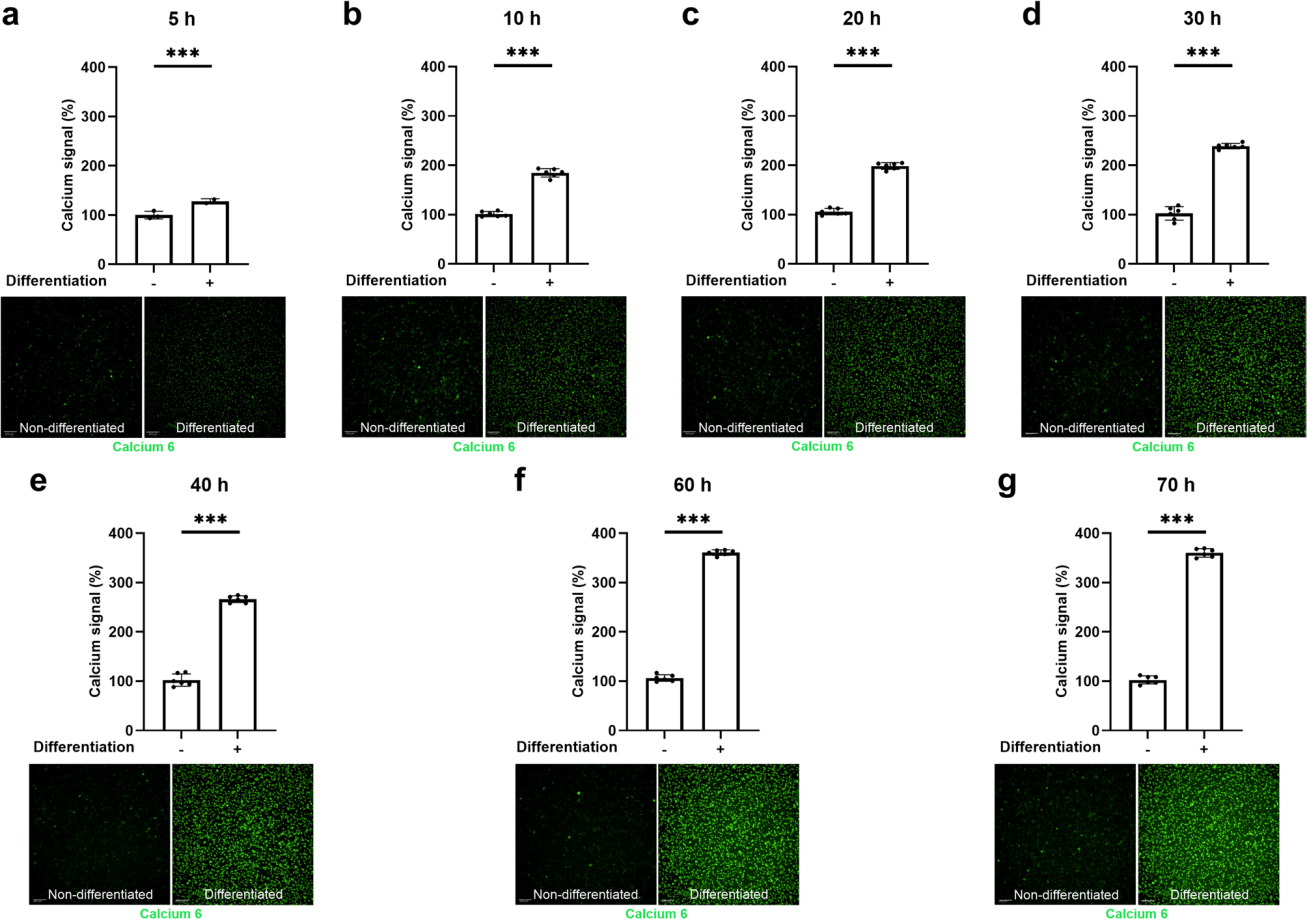
Differentiation increased the basal intracellular calcium levels in F11 cells compared to non-differentiated F11 cells. Calcium levels (mean ± SD) and representative images marking intracellular calcium (green) comparing non-differentiated (−) and differentiated F11 cells (+) after **a** 5 h, **b** 10 h, **c** 20 h, **d** 30 h, **e** 40 h, **f** 60 h and **g** 70 h. Data and images are representative of one experiment out of three independent experiments (*N* = 3). Each data point represents one replicate within the experiment (*n* = 6). ***p < 0.001; Welch’s *t* test. Values are expressed as a percentage relative to the intensity observed in non-differentiated F11 cells, which is set as 100%. Scale bar in microphotographs = 200 μm.

Differentiation of F11 cells was associated with a sustained increase in basal intracellular calcium levels (*p* < 0.001) (Fig. 2). Basal intracellular Ca²⁺ levels increased over differentiation and appeared to plateau from 60 h onward (reaching ~ 360% of non-differentiated F11 cell values); therefore, and in line with established F11 differentiation protocols^15^, we used 72 h as the standard endpoint for subsequent functional and imaging analyses.

**Fig. 3:**
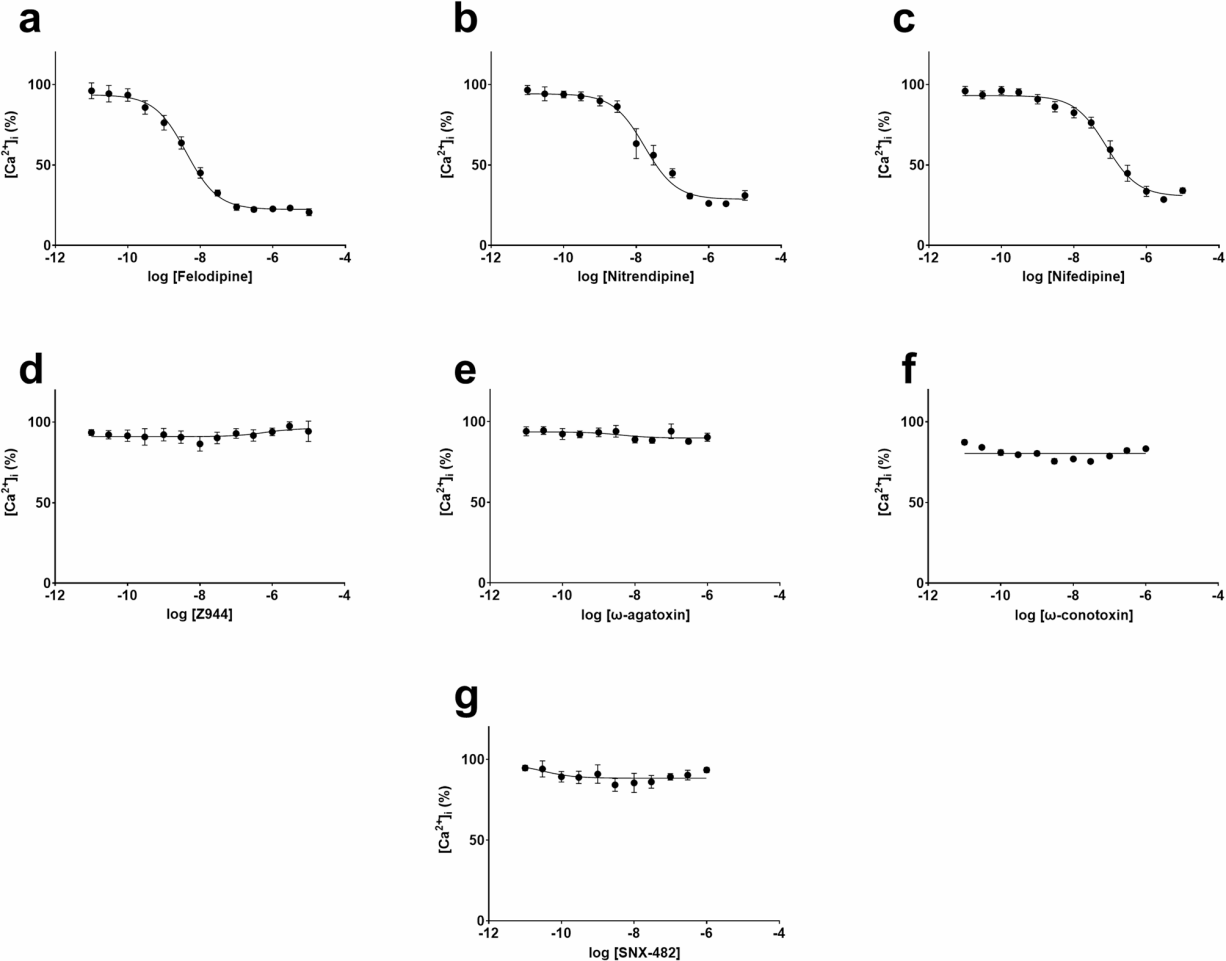
1,4-dihydropyridines during differentiation reduced KCl-evoked intracellular Ca^2+^ signals in differentiated F11 cells. Effect of serial concentrations of 1,4-dihydropyridines **a** felodipine, **b** nitrendipine and **c** nifedipine added throughout the 72 h differentiation period on 30 mM KCl-evoked intracellular Ca^2+^ signals in F11 cells. Effect of serial concentrations of **d** Z944, **e** ω-agatoxin, **f** ω-conotoxin and **g** SNX-482 present during the 72 h differentiation period on 30 mM KCl-evoked intracellular Ca^2+^ signals. Results are presented as mean ± S.D. from three independent experiments (*N* = 3), each with two replicates per experiment (*n* = 2). Values are expressed as a percentage relative to the intracellular Ca^2+^ signal observed in vehicle-differentiated F11 cells not exposed to any VGCC inhibitor, which is set as 100%.

To determine the specific contribution of each VGCC type to the acquisition of KCl-evoked intracellular Ca^2+^ signals during differentiation, F11 cells were differentiated for 72 h in the presence of selective antagonists targeting different calcium channel types and then challenged with 30 mM KCl. Exposure to 1,4-dihydropyridines (nitrendipine, felodipine, and nifedipine), which selectively inhibit L-type VGCCs, throughout the differentiation period at concentrations ranging from 10 pM to 10 µM resulted in a significant reduction in the KCl-evoked intracellular Ca²⁺ signal at 72 h compared to vehicle-differentiated controls (Fig. 3a–c) (S2 Fig, panel b-d). Exposure to felodipine, nitrendipine and nifedipine throughout the 72 h differentiation period led to a concentration-dependent inhibition of the 30 mM KCl-evoked intracellular Ca²⁺ signal measured at 72 h, with pIC_50_ values of 8.39 ± 0.07, 7.77 ± 0.09 and 7.11 ± 0.08, corresponding to IC_50_ values of approximately 4, 17 and 77 nM, respectively (Table 1). In contrast, differentiation in the presence of Z944 at concentrations ranging from 10 pM to 10 µM or Ca_V_2 channel blockers (ω-agatoxin, ω-conotoxin, or SNX-482) at concentrations ranging from 10 pM to 1 µM did not significantly alter the KCl-evoked intracellular Ca²⁺ signal measured after 72 h (Fig. 3d–g), despite the upregulation of Ca_V_2-encoding genes (*Cacna1a*, *Cacna1b* and *Cacna1e*) upon differentiation (Fig. 1b–c). These findings support that L-type VGCC activity is required for F11 cells to acquire KCl-evoked intracellular Ca²⁺ responsiveness as they develop neuronal features during differentiation.

**Table 1:** Potency (IC₅₀) and efficacy (I_max_) of calcium channel blockers in inhibiting 30 mM KCl-evoked intracellular Ca²⁺ signals in differentiated F11 cells after 72 h of exposure during the differentiation period.

Compound	IC_50_ (nM, mean ± S.D.)	I_max_ (%) (% inhibition of KCl-evoked intracellular Ca²⁺ signal; mean ± S.D.)
Felodipine	4.10 ± 0.64	76.63 ± 1.81
Nitrendipine	17.4 ± 3.72	74.27 ± 1.11
Nifedipine	78.5 ± 14.1	69.51 ± 1.26
Z944	–	9.03 ± 1.29
ω-agatoxin	–	10.14 ± 1.57
ω-conotoxin	–	20.21 ± 0.64
SNX-482	–	11.64 ± 0.69

**Fig. 4:**
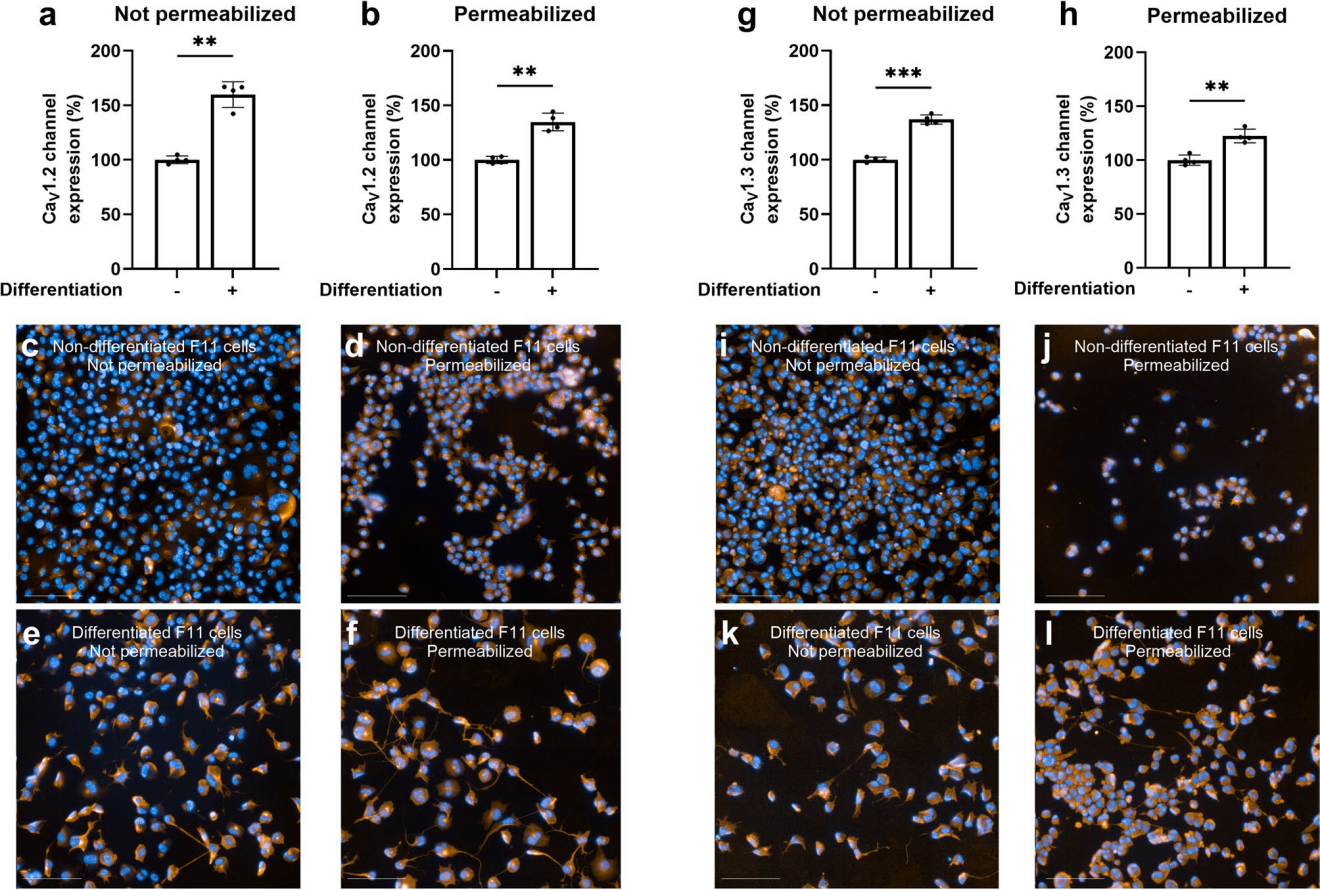
Differentiation of F11 cells increased the expression of Ca_V_1.2 and Ca_V_1.3 VGCCs. Expression of Ca_V_1.2 VGCCs in **a** the cell membrane (non-permeabilized cells) and **b** the whole cell (permeabilized cells) of non-differentiated (–) and differentiated F11 cells (+) (mean ± S.D.). **c**–**f** Representative images of non-differentiated and differentiated F11 cells stained for nuclei (blue) and Ca_V_1.2 VGCCs (orange) under non-permeabilized and permeabilized conditions. Expression of Ca_V_1.3 VGCCs in **g** the cell membrane (non-permeabilized cells) and **h** the whole cell (permeabilized cells) of non-differentiated (–) and differentiated F11 cells (+) (mean ± S.D.). **i**–**l** Representative images of non-differentiated and differentiated F11 cells stained for nuclei (blue) and Ca_V_1.3 VGCCs (orange) under non-permeabilized and permeabilized conditions. Data and images for both channels are representative of one experiment out of three independent experiments (*N* = 3). Each data point represents one replicate within the experiment (*n* = 4). Values in each graph are expressed as a percentage relative to the expression of Ca_V_1.2 or Ca_V_1.3 VGCCs, respectively, in non-differentiated F11 cells in each condition, which is defined as 100%. ***p < 0.001, **p < 0.01; Welch’s *t* test. Scale bar in microphotographs = 100 μm.

This reduction in KCl-evoked intracellular Ca^2+^ signals in F11 cells following exposure to selective L-type VGCC antagonists during differentiation aligns with the significant upregulation of genes encoding the DRG neuron-associated L-type VGCCs, Ca_V_1.2 (*Cacna1c*) and Ca_V_1.3 (*Cacna1d*), as noted above, during differentiation (Fig. 1b–c). These findings are further supported by the increased expression of Ca_V_1.2 and Ca_V_1.3 VGCCs both at the plasma membrane and at the whole-cell level in differentiated F11 cells compared to non-differentiated cells (*p* < 0.01) (Fig. 4). In addition, compartmental analysis showed a preferential somatic enrichment of Ca_V_1.3 relative to neurites, whereas Ca_V_1.2 displayed a more uniform soma–neurite distribution (S4 Fig). Together, these observations indicate a greater availability of L-type channels to contribute to the KCl-evoked intracellular Ca²⁺ signals associated with differentiation. Altogether, these results underscore the relevance of Ca_V_1.2 and Ca_V_1.3 VGCCs in the differentiation of F11 cells.

**Fig. 5:**
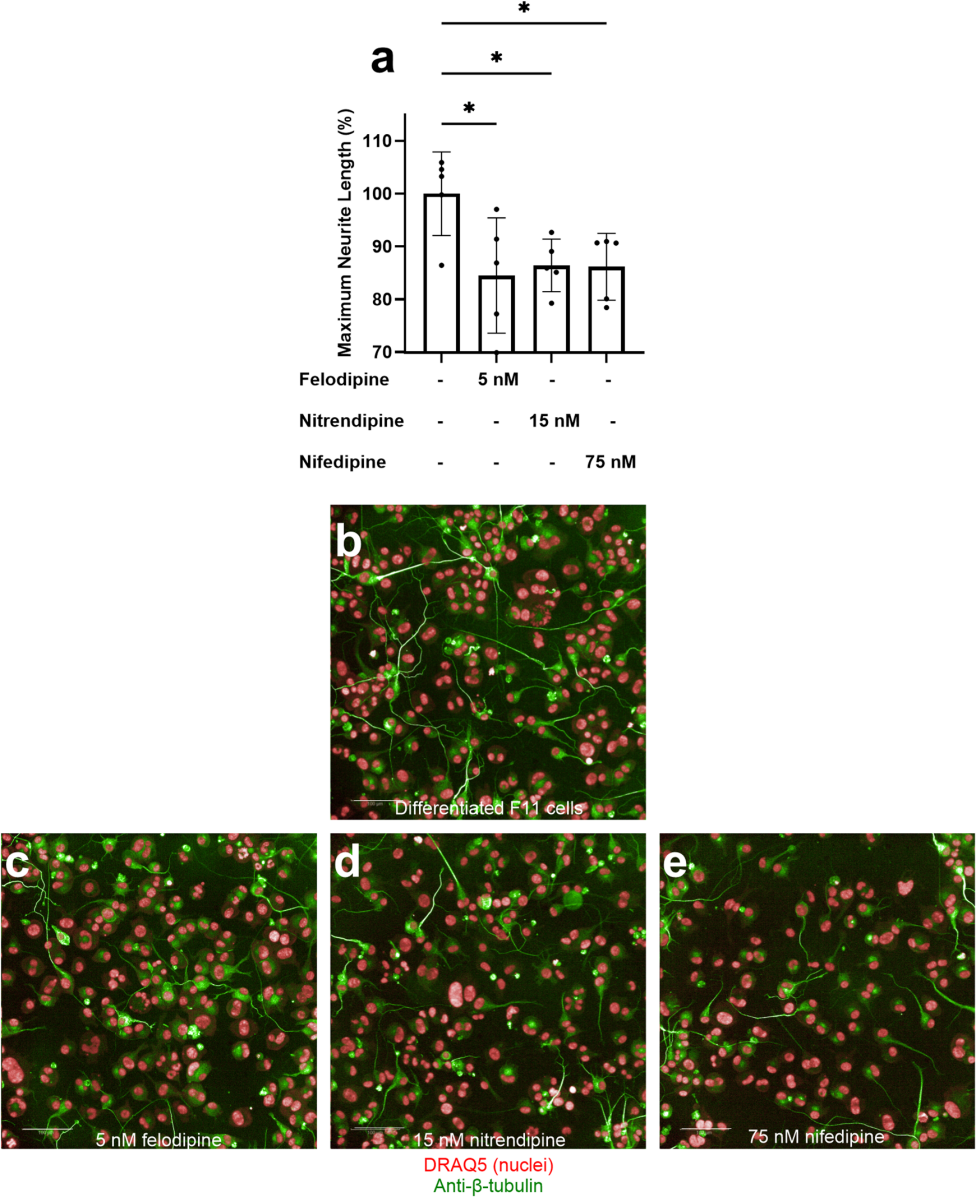
Exposure to 1,4-dihydropyridines during differentiation impaired neurite outgrowth. **a** Effect of 5 nM felodipine, 15 nM nitrendipine and 75 nM nifedipine on maximum neurite length during differentiation, compared to control differentiated F11 cells (mean ± S.D.). Each data point represents one replicate within the experiment (*n* = 5). Values are expressed as a percentage relative to the maximum neurite length of control differentiated F11 cells not exposed to any 1,4-dihydropyridine, which is set as 100%. *p < 0.05; ANOVA followed by Dunnett’s post-hoc analysis. Representative images of **b** control differentiated F11 cells and of F11 cells exposed to **c** 5 nM felodipine, **d** 15 nM nitrendipine and **e** 75 nM nifedipine stained for nuclei (red) and β-tubulin (green). Scale bar = 100 μm. Data and images are representative of one experiment out of three independent experiments (*N* = 3).

With respect to the effect of 1,4-dihydropyridines as selective inhibitors of L-type VGCCs on neurite outgrowth during F11 cell differentiation, exposure to 5 nM felodipine, 15 nM nitrendipine, and 75 nM nifedipine, concentrations approximating their respective IC₅₀ values for inhibition of the 30 mM KCl-evoked intracellular Ca²⁺ signal, resulted in a significant reduction in neurite outgrowth (*p* < 0.05) (Fig. 5), without compromising the viability of differentiated F11 cells compared to control cells (S5 Fig). These findings confirm that L-type VGCC–dependent KCl-evoked intracellular Ca^2+^ signals play a critical role in the acquisition of neuronal features during the differentiation process of F11 cells.

### Transfection with VGCC-encoding genes promoted the acquisition of neuronal features in non-differentiated F11 cells under basal conditions but impaired this acquisition during differentiation related to an increase in oxidative stress

We investigated the effects of *Cacna1c* and *Cacna1d* gene overexpression, which encode the Ca_V_1.2 and Ca_V_1.3 channels, respectively, through plasmid transfection on F11 cells under both basal and differentiation conditions.

**Fig. 6:**
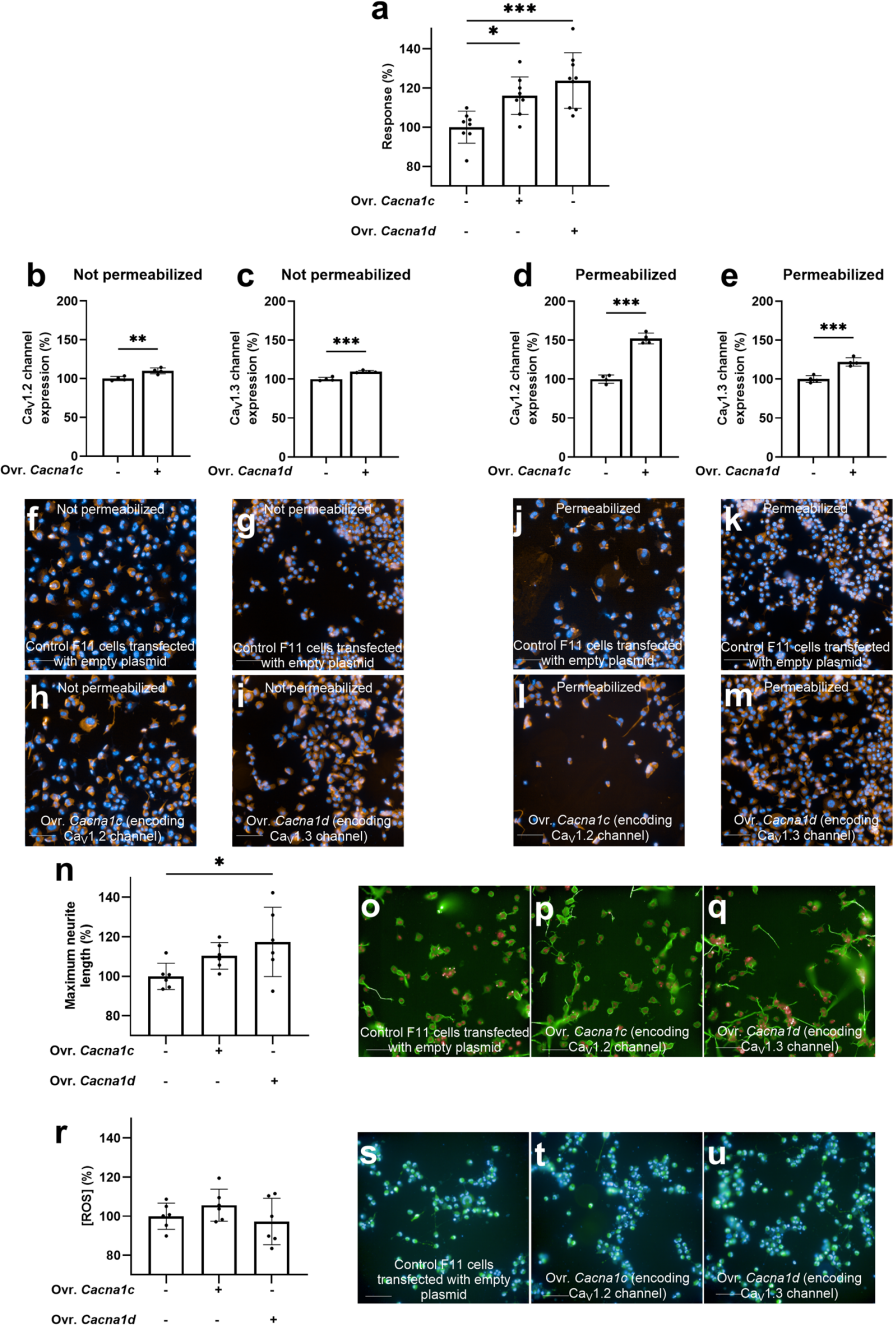
Ca_V_1.3 overexpression enhanced neurite outgrowth under basal conditions, while Ca_V_1.2 and Ca_V_1.3 increased the 30 mM KCl-evoked intracellular Ca²⁺ signal. **a** Effect of 30 mM KCl on intracellular calcium signal in F11 cells after overexpression (Ovr.) of Ca_V_1.2 and Ca_V_1.3 channels compared to control F11 cells (mean ± S.D.). Data are representative of one experiment out of three independent experiments (*N* = 3). Each data point represents one replicate within the experiment (*n* = 9). ***p < 0.001, *p < 0.05; ANOVA followed by Dunnett’s post-hoc analysis. Effect of the overexpression of Ca_V_1.2 and Ca_V_1.3 under basal conditions in **b**, **c** not permeabilized and **d**, **e** permeabilized cells. Data are representative of one experiment out of three independent experiments (*N* = 3). Each data point represents one replicate within the experiment (*n* = 4). ***p < 0.001, **p < 0.01; Welch’s *t* test. **f**–**m** Representative images of each condition with nuclei stained in blue and channels stained in orange. **n** Effect of overexpression of Ca_V_1.2 and Ca_V_1.3 channels on maximum neurite length compared to control F11 cells (mean ± S.D). Data are representative of one experiment out of three independent experiments (*N* = 3). Each data point represents one replicate within the experiment (*n* = 6). *p < 0.05; ANOVA followed by Dunnett’s post-hoc analysis. Representative images of **o** control F11 cells and F11 cells after overexpression of **p** Ca_V_1.2 channels and **q** Ca_V_1.3 channels stained for nuclei (red) and β-tubulin (green). **r** Effect of overexpression of Ca_V_1.2 and Ca_V_1.3 channels on CellROX Green intensity, representing intracellular ROS concentrations, compared to control F11 cells (mean ± S.D). Data are representative of one experiment out of four independent experiments (*N* = 4). Each data point represents one replicate within the experiment (*n* = 6). Representative images of **s** control F11 cells and F11 cells after overexpression of **t** Ca_V_1.2 and **u** Ca_V_1.3 channels stained for nuclei (blue) and with CellROX Green. Values in graphs are expressed as a percentage relative to the effect observed in control F11 cells transfected with an empty plasmid (100%). Scale bar in microphotographs = 100 μm.

Overexpression of either the Ca_V_1.2 or the Ca_V_1.3 channels under basal conditions (i.e., culture medium with reduced FBS concentration) significantly increased the 30 mM KCl-evoked intracellular Ca^2+^ signal compared to F11 cells transfected with an empty vector (*p* < 0.05) (Fig. 6a) (S3 Table) (S2 Fig, panel e). This functional increase was accompanied by higher channel expression after transfection, both at the plasma membrane and at the whole-cell level (*p* < 0.01) (Fig. 6b–m). However, only Ca_V_1.3 channels overexpression resulted in a significant 17% increase in maximum neurite length (*p* < 0.05), while no significant changes in maximum neurite length were observed after Ca_V_1.2 channels overexpression (*p* = 0.24) (Fig. 6n–q) (S3 Table).

Given the established link between sustained L-type Ca²⁺ influx and oxidative stress, we measured intracellular ROS to assess whether Ca_V_1.2/Ca_V_1.3 overexpression alters redox homeostasis under basal conditions. No changes in intracellular ROS concentrations were observed after overexpression of either Ca_V_1.2 or Ca_V_1.3 channels under basal conditions (Fig. 6r–u). These results support an important role for L-type VGCC–dependent KCl-evoked intracellular Ca²⁺ signals in F11 cell differentiation and indicate that Ca_V_1.3 channels have a more pronounced impact than Ca_V_1.2 channels on the acquisition of neuronal features, particularly neurite outgrowth.

**Fig. 7:**
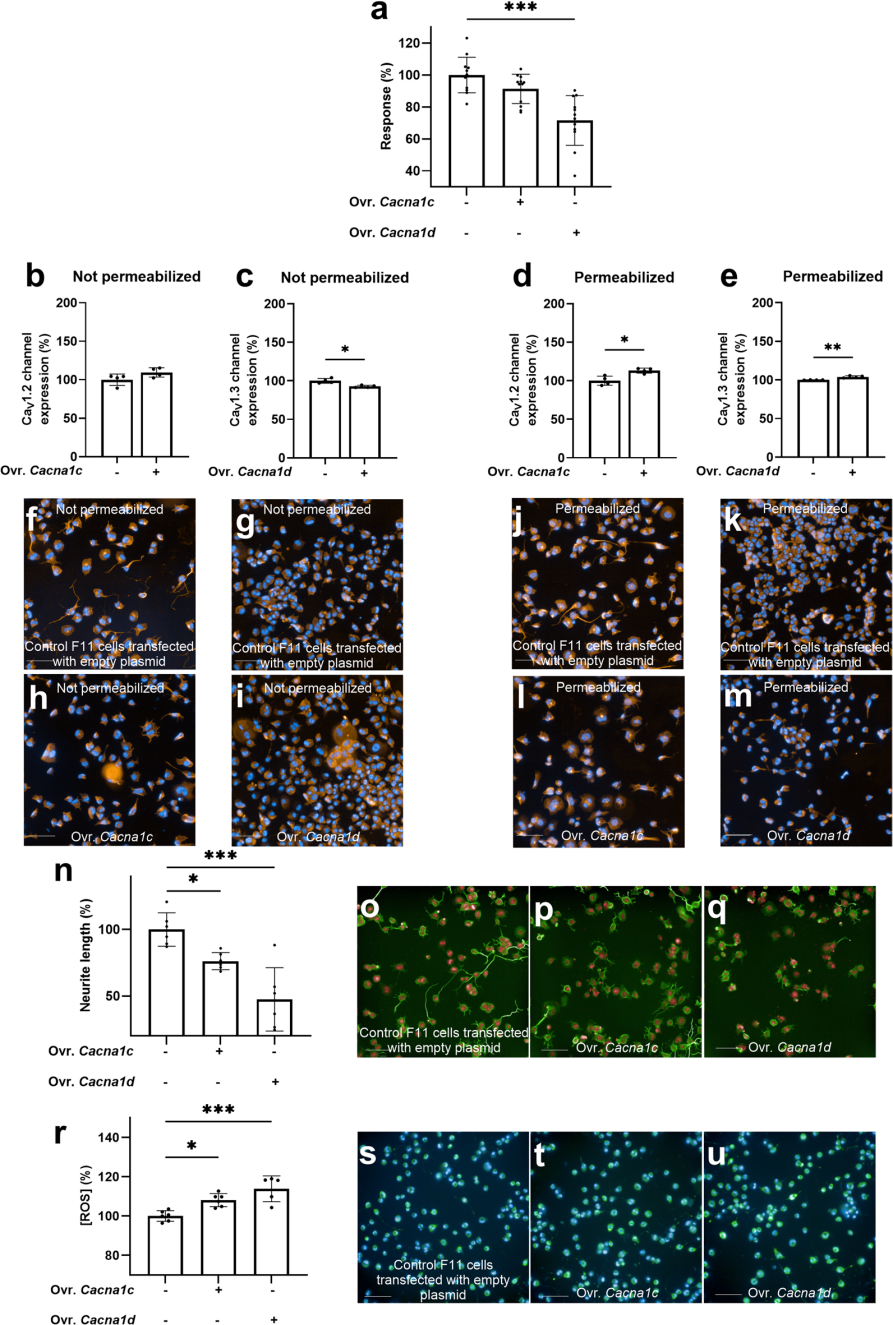
Ca_V_1.2 and Ca_V_1.3 overexpression under differentiation conditions impaired acquisition of neuronal features and increased ROS. **a** Effect of 30 mM KCl on intracellular calcium signal in F11 cells after overexpression (Ovr.) of Ca_V_1.2 and Ca_V_1.3 channels compared to control F11 cells (mean ± S.D.). Data are representative of one experiment out of three independent experiments (*N* = 3). Each data point represents one replicate within the experiment (*n* = 9). ***p < 0.001; ANOVA followed by Dunnett’s post-hoc analysis. Effect of the overexpression of Ca_V_1.2 and Ca_V_1.3 under differentiation conditions in **b**, **c** not permeabilized and **d**, **e** permeabilized cells. Data are representative of one experiment out of three independent experiments (*N* = 3). Each data point represents one replicate within the experiment (*n* = 4). **p < 0.01, *p < 0.05; Welch’s *t* test. **f**–**m** Representative images of each condition with nuclei stained in blue and channels stained in orange. **n** Effect of overexpression of Ca_V_1.2 and Ca_V_1.3 channels on maximum neurite length compared to control F11 cells (mean ± S.D). Data are representative of one experiment out of three independent experiments (*N* = 3). Each data point represents one replicate within the experiment (*n* = 6). ***p < 0.001, *p < 0.05; ANOVA followed by Dunnett’s post-hoc analysis. Representative images of **o** control F11 cells and F11 cells after overexpression of **p** Ca_V_1.2 channels and **q** Ca_V_1.3 channels stained for nuclei (red) and β-tubulin (green). **r** Effect of overexpression of Ca_V_1.2 and Ca_V_1.3 channels on CellROX Green intensity, representing intracellular ROS concentrations, compared to control F11 cells (mean ± S.D). Data are representative of one experiment out of four independent experiments (*N* = 4). Each data point represents one replicate within the experiment (*n* = 6). ***p < 0.001, *p < 0.05; ANOVA followed by Dunnett’s post-hoc analysis. Representative images of (**s**) control F11 cells and F11 cells after overexpression of **t** Ca_V_1.2 and **u** Ca_V_1.3 channels stained for nuclei (blue) and with CellROX Green. Values in graphs are expressed as a percentage relative to the effect observed in control F11 cells transfected with an empty plasmid (100%). Scale bar in microphotographs = 100 μm.

In F11 cells under differentiation conditions (i.e., in differentiation medium), overexpression of Ca_V_1.3 channels resulted in a 28% lower 30 mM KCl-evoked intracellular Ca²⁺ signal compared to F11 cells transfected with an empty vector (*p* < 0.001) (Fig. 7a) (S3 Table) (S2 Fig, panel f), in line with a reduction in the expression of the channel in the membrane of F11 cells (Fig. 7c, g, i) but an increase in whole-cell (*p* < 0.01) (Fig. 7e, k, m). In contrast, Ca_V_1.2 channels overexpression did not result in significant changes in the 30 mM KCl-evoked intracellular Ca²⁺ signal (*p* = 0.16), consistent with the lack of changes in the expression of the channel in the membrane (*p* = 0.1) (Fig. 7b, f, h) but an increase in whole-cell (*p* < 0.05) (Fig. 7d, j, l). Additionally, overexpression of both channels in F11 cells under differentiation conditions resulted in a decrease in neurite outgrowth, with a 24% reduction following Ca_V_1.2 channels overexpression (*p* < 0.05) and a 52% reduction after Ca_V_1.3 channels overexpression (*p* < 0.001), compared to control F11 cells (Fig. 7n-q) (S3 Table). Furthermore, overexpression of any of the channels under differentiation conditions elicited a significant increase in intracellular ROS levels (Fig. 7r-u), with an 8.1% increase following Ca_V_1.2 channels overexpression (*p* < 0.05) and a 13.9% increase following Ca_V_1.3 channels overexpression (*p* < 0.001) (S3 Table). These findings underscore the involvement of L-type VGCCs in both the differentiation of F11 cells and oxidative stress, emphasizing the need for tight regulation of VGCC expression, particularly Ca_V_1.3, to ensure the acquisition of neuronal phenotypic features during differentiation.

## Discussion

In this study we identify L-type Ca_V_1.3 voltage-gated calcium channels as key regulators of the differentiation program of the immortalized dorsal root ganglion (DRG)–derived F11 cell line. VGCCs are highly expressed in DRG neurons and contribute to both developmental processes and pain signaling pathways^23–26^. While previous work from our group established a role for Na_V_1.5-mediated sodium transients in F11 cell differentiation^17^, the present work provides, to our knowledge, the first integrated genomic and pharmacological analysis of calcium channel involvement in the differentiation of an immortalized neuronal cell line.

During differentiation, F11 cells acquired core neuronal phenotypic features, including neurite outgrowth and an increase in depolarization-evoked intracellular Ca^2+^ in response to 30 mM KCl^12^, accompanied by a sustained elevation in basal cytoplasmic Ca^2+^ levels and increased spontaneous spiking activity measured by multielectrode arrays. Consistent with this, differentiation robustly upregulated transcripts encoding multiple VGCC α subunits. We observed increased expression of *Cacna1a*, *Cacna1b*, and *Cacna1e* and of *Cacna1g–i*, which encode Ca_V_2 and Ca_V_3 channels that are well documented in DRG neurons and contribute to synaptic transmission and pain processing^27–29^. By contrast, *Cacna1s* and *Cacna1f*, encoding skeletal muscle Ca_V_1.1 and retinal Ca_V_1.4 channels^21,28^, remained undetectable, while *Cacna1c* and *Cacna1d*, encoding the L-type Ca_V_1.2 and Ca_V_1.3 channels that are widely expressed in DRG neurons^30,31^, were markedly upregulated. This convergence between the VGCC transcriptomic profile of differentiated F11 cells and that described for DRG neurons is consistent with the view that F11 cells recapitulate selected DRG-like features, supporting their use as an in vitro system to probe sensory neuron–relevant mechanisms.

Selective pharmacological blockade further pointed to a dominant contribution of L-type channels to the KCl-evoked Ca^2+^ signal associated with differentiation. Neither the T-type blocker Z944 nor the P/Q-, N- or R-type toxins altered the 30 mM KCl-evoked Ca^2+^ response after 72 h of exposure, despite clear upregulation of the corresponding Ca_V_2 and Ca_V_3 transcripts. In contrast, 1,4-dihydropyridines produced a concentration-dependent inhibition of the KCl-evoked Ca^2+^ signal, with felodipine being more potent than nitrendipine and nifedipine. The apparent functional IC_50_ values for inhibition of the KCl-evoked Ca^2+^ signal (4, 17 and 78 nM, respectively) should be interpreted as long-term potencies in a differentiation context, rather than as biophysical measures of unitary L-type current block. Nevertheless, the rank order of potency parallels clinical data in humans, in which felodipine induces reflex tachycardia at lower plasma concentrations than nitrendipine or nifedipine^32^, and aligns with the increased membrane expression of Ca_V_1.2 and Ca_V_1.3 in differentiated F11 cells. Together with the observation that dihydropyridine exposure at concentrations approximating these IC_50_ values reduces neurite outgrowth without affecting viability, these findings support a causal role for L-type channel–mediated Ca^2+^ signals in the acquisition of neuronal phenotypic features in this model.

Our gain-of-function experiments with Ca_V_1.2 and Ca_V_1.3 provide further insight into how L-type channel activity shapes differentiation. Under basal, low-serum conditions, overexpression of either channel increased the KCl-evoked Ca^2+^ signal. This was consistent with the increased channel signal observed after transfection at both the plasma membrane and the whole-cell level, supporting enhanced functional availability of L-type channels. However, only CaV1.3 VGCCs overexpression significantly promoted neurite outgrowth, consistent with previous findings by Marschallinger et al. (2015), who identified a critical role of Ca_V_1.3 in hippocampal neurogenesis^33^. This is also in line with Zhang et al. (2006), who showed in hippocampal neurons that Ca_V_1.3 VGCCs couple more efficiently than Ca_V_1.2 to CREB phosphorylation^34^. Given the established role of CREB in neuronal development and F11 differentiation^16,35^, these observations are consistent with the possibility that Ca_V_1.3, more than Ca_V_1.2, may preferentially engage CREB-dependent pathways involved in neurite outgrowth in this model.

In contrast, when Ca_V_1.2 or Ca_V_1.3 were overexpressed under differentiation conditions, both channels reduced neurite length and increased intracellular ROS, with Ca_V_1.3 having the more pronounced effect. Notably, under differentiation conditions, overexpression did not translate into higher plasma membrane channel signal (and for Ca_V_1.3 it was reduced) despite increased whole-cell signal, indicating a differentiation-dependent uncoupling between total channel abundance and surface-associated channel availability. Ca_V_1.3 activates at more hyperpolarized potentials and can sustain prolonged Ca^2+^ entry^18,36^, a biophysical profile that is compatible with enhanced vulnerability to oxidative stress under differentiation conditions, when overall Ca^2+^ load is already elevated. Our data are therefore consistent with a dual role for L-type channels in this system: moderate Ca_V_1.3-dependent Ca^2+^ signaling may favor the acquisition of neuronal traits, whereas higher levels of L-type expression in an already Ca^2+^-loaded differentiating milieu, consistent with altered surface availability, may contribute to increased oxidative stress and impaired differentiation. Similar dissociations between total and surface expression of L-type channels have been reported in neurons, linked to auxiliary subunit availability and activity-dependent internalization^37,38^.

Several methodological considerations are important when interpreting these results. All Ca²⁺ measurements were obtained by population-based optical imaging of intracellular Ca²⁺ with FLIPR Calcium-6 after depolarization with 30 mM KCl. This format provides a robust, high-throughput readout of depolarization-evoked Ca²⁺ signals in large F11 cell populations, well suited to phenotypic interrogation of VGCC-dependent signaling^39^. In parallel, multielectrode array recordings provided convergent evidence of differentiation-associated increases in spontaneous spiking activity at the population level (S3 Fig). KCl-evoked responses in this setting are not intended to provide direct measurements of membrane potential dynamics or unitary currents through individual VGCC subtypes; instead, they reflect the integrated contribution of Ca²⁺ entry via VGCCs together with Ca²⁺-induced Ca²⁺ release from intracellular stores^40,41^. We therefore interpret the observed changes as integrated functional readouts of L-type channel–dependent Ca²⁺ entry within the broader Ca²⁺ signaling machinery of F11 cells, while not ruling out a contribution from intracellular stores. Importantly, our previous work using the same differentiation protocol showed reduced SOCE and downregulation of *Trpc3* and *Trpc5*^17^, making it unlikely that an increased store-operated component is the primary driver of the larger KCl-evoked Ca²⁺ signals observed here.

Beyond these technical aspects, it is also important to place the F11 model in the appropriate biological context relative to primary human DRG neurons. F11 cells are a mouse neuroblastoma × rat embryonic DRG hybrid line that provides a stable, reductionist system capturing key DRG-like properties and enabling reproducible mechanistic dissection of VGCC-dependent pathways^15^. At the same time, they do not encompass the full cellular and molecular diversity of human DRG tissue: F11 cells are maintained in 2D monoculture, without satellite glial or immune cells or the native extracellular matrix and vascular milieu that influence excitability and survival in vivo, and they cannot fully recapitulate the heterogeneity of human nociceptor subtypes^12,42^. Species-specific differences in channel splice variants, accessory subunits and signaling networks are also likely. Consequently, the quantitative relationships between VGCC expression, Ca²⁺ dynamics and neurite outgrowth described here are best viewed as mechanistic within this well-defined model, providing a framework to guide and prioritize complementary studies in primary rodent or human DRG cultures and in human iPSC-derived sensory neurons.

Finally, although our work focuses on VGCCs, F11 differentiation is likely regulated by additional pathways. The differentiation protocol relies on cAMP-elevating agents that converge on CREB activation, and prior work in F11 cells has highlighted contributions from voltage-gated sodium channels, neurotrophins and other growth factors to the acquisition of neuronal phenotypic features^17,43,44^. Our findings should therefore be viewed as defining a critical node in the differentiation process, namely L-type Ca²⁺ entry and its redox consequences, within a broader network of signaling mechanisms that collectively govern the differentiation process.

In summary, we provide a comprehensive characterization of VGCC involvement in F11 cell differentiation, highlighting a central role for L-type Ca_V_1.2 and especially Ca_V_1.3 channels. Through combined transcriptomic, pharmacological and genetic approaches we show that L-type channel–dependent KCl-evoked Ca^2+^ signals are tightly associated with the acquisition of neuronal phenotypic features, and that excessive Ca_V_1.3 activity in a high-Ca^2+^ context promotes oxidative stress and impairs differentiation. While the immortalized F11 model has inherent limitations relative to primary human DRG neurons, it offers a tractable platform for high-content, Ca^2+^-based phenotypic assays that incorporate VGCC biology into early-stage drug discovery for neuropathic pain and neurodegenerative diseases.

## Supplementary Information

Below is the link to the electronic supplementary material.

Supplementary Material 1

## Data Availability

All data generated or analyzed during this study are included in this published article and its Supplementary Information file.

## References

[1] Setien, M. B. et al. Differentiation and characterization of neurons derived from rat iPSCs. *J. Neurosci. Methods* **338**, 108693 (2020). 32199944 10.1016/j.jneumeth.2020.108693 PMC8883348

[2] Toth, A. B., Shum, A. K. & Prakriya, M. Regulation of neurogenesis by calcium signaling. *Cell. Calcium*. **59**, 124–134 (2016). 27020657 10.1016/j.ceca.2016.02.011 PMC5228525

[3] Heck, J. et al. More than a pore: How voltage-gated calcium channels act on different levels of neuronal communication regulation. *Channels* **15**, 322–338 (2021). 34107849 10.1080/19336950.2021.1900024 PMC8205089

[4] Kamijo, S. et al. A critical neurodevelopmental role for L-type voltage-gated calcium channels in neurite extension and radial migration. *J. Neurosci.* **38**, 5551–5566 (2018). 29773754 10.1523/JNEUROSCI.2357-17.2018 PMC8174135

[5] Dolmetsch, R. E., Pajvani, U., Fife, K., Spotts, J. M. & Greenberg, M. E. Signaling to the nucleus by an L-type calcium channel-calmodulin complex through the MAP kinase pathway. *Science (1979)* **294**, 333–339 (2001). 10.1126/science.1063395 11598293

[6] Bourinet, E. et al. Calcium-permeable ion channels in pain signaling. *Physiol. Rev.* **94**, 81–140 (2014). 24382884 10.1152/physrev.00023.2013

[7] Schampel, A., Kuerten, S. Danger: high voltage—the role of voltage-gated calcium channels in central nervous system pathology. *Cells* **6**, 43 (2017). 29140302 10.3390/cells6040043 PMC5755501

[8] Bourinet, E., Francois, A. & Laffray, S. T-type calcium channels in neuropathic pain. *Pain* **157**, S15–S22 (2016). 26785151 10.1097/j.pain.0000000000000469

[9] Yaksh, T. L. Calcium channels as therapeutic targets in neuropathic pain. *Journal Pain* **7**, S13-S30 (2006). 10.1016/j.jpain.2005.09.007 16426997

[10] Cataldi, M. The changing landscape of voltage-gated calcium channels in neurovascular disorders and in neurodegenerative diseases. *Curr. Neuropharmacol.* **11**, 276–297 (2013). 24179464 10.2174/1570159X11311030004 PMC3648780

[11] Alles, S. R. A. & Smith, P. A. Peripheral voltage-gated cation channels in neuropathic pain and their potential as therapeutic targets. *Front. Pain Res.* **2**, 750583 (2021). 10.3389/fpain.2021.750583 PMC8915663 35295464

[12] Martínez, A. L. et al. In vitro models for neuropathic pain phenotypic screening in brain therapeutics. *Pharmacol. Res.* **202**, 107111 (2024). 38382648 10.1016/j.phrs.2024.107111

[13] Martínez, A. L. et al. A new model of sensorial neuron-like cells for HTS of novel analgesics for neuropathic pain. *SLAS Discov.* **24**, 158–168 (2019). 30383474 10.1177/2472555218810323

[14] Hashemian, S., Alhouayek, M. & Fowler, C. J. TLR4 receptor expression and function in F11 dorsal root ganglion × neuroblastoma hybrid cells. *Innate Immun.* **23**, 687–696 (2017). 28958207 10.1177/1753425917732824

[15] Haberberger, R. V., Barry, C. & Matusica, D. Immortalized Dorsal Root Ganglion Neuron Cell Lines. *Front. Cell. Neurosci.* **14**, 184 (2020). 32636736 10.3389/fncel.2020.00184 PMC7319018

[16] Ghil, S. H., Kim, B. J., Lee, Y. D. & Suh-Kim, H. Neurite outgrowth induced by cyclic AMP can be modulated by the α subunit of Go. *J. Neurochem*. **74**, 151–158 (2001). 10.1046/j.1471-4159.2000.0740151.x 10617116

[17] Martínez, A. L. et al. Identification of sodium transients through NaV1.5 channels as regulators of differentiation in immortalized dorsal root ganglia neurons. *Front. Cell. Neurosci.* **16**, 816325 (2022). 35465610 10.3389/fncel.2022.816325 PMC9018981

[18] Xu, W. & Lipscombe, D. Neuronal Ca1.3α1 L-type channels activate at relatively hyperpolarized membrane potentials and are incompletely inhibited by dihydropyridines. *J. Neurosci.* **21**, 5944–5951 (2001). 11487617 10.1523/JNEUROSCI.21-16-05944.2001 PMC6763157

[19] Helton, T. D., Xu, W. & Lipscombe, D. Neuronal L-type calcium channels open quickly and are inhibited slowly. *J. Neurosci.* **25**, 10247–10251 (2005). 16267232 10.1523/JNEUROSCI.1089-05.2005 PMC6725800

[20] McKerr, N. et al. CACNA1D overexpression and voltage-gated calcium channels in prostate cancer during androgen deprivation. *Sci. Rep.* **13**, 4683 (2023). 36949059 10.1038/s41598-023-28693-y PMC10033880

[21] Mansergh, F. et al. Mutation of the calcium channel gene Cacna1f disrupts calcium signaling, synaptic transmission and cellular organization in mouse retina. *Hum. Mol. Genet.* **14**, 3035–3046 (2005). 16155113 10.1093/hmg/ddi336

[22] Dolphin, A. C. Voltage-gated calcium channels and their auxiliary subunits: physiology and pathophysiology and pharmacology. *J. Physiol.* **594**, 5369–5390 (2016). 27273705 10.1113/JP272262 PMC5043047

[23] Hackney, C. M. et al. A previously unrecognized superfamily of macro-conotoxins includes an inhibitor of the sensory neuron calcium channel Cav2.3. *PLoS Biol.* **21**, e3002217 (2023). 37535677 10.1371/journal.pbio.3002217 PMC10437998

[24] Cai, Z., Quan, L., Chang, X., Qiu, Z. & Zhou, H. High-voltage long-duration pulsed radiofrequency attenuates neuropathic pain in CCI rats by inhibiting Cav2.2 in spinal dorsal horn and dorsal root ganglion. *Brain Res.* **1785**, 147892 (2022). 35341732 10.1016/j.brainres.2022.147892

[25] Mintz, I. M., Adams, M. E. & Bean, B. P. P-type calcium channels in rat central and peripheral neurons. *Neuron* **9**, 85–95 (1992). 1321648 10.1016/0896-6273(92)90223-z

[26] Hartung, J. E. et al. Voltage-gated calcium currents in human dorsal root ganglion neurons. *Pain* **163**, e774–e785 (2022). 34510139 10.1097/j.pain.0000000000002465 PMC8882208

[27] Ferreira, M. A. et al. Sex-dependent Cav2.3 channel contribution to the secondary hyperalgesia in a mice model of central sensitization. *Brain Res.* **1764**, 147438 (2021). 33753067 10.1016/j.brainres.2021.147438

[28] Simms, B. A. & Zamponi, G. W. Neuronal voltage-gated calcium channels: structure, function, and dysfunction. *Neuron* **82**, 24–45 (2014). 24698266 10.1016/j.neuron.2014.03.016

[29] François, A. et al. T-type calcium channels in chronic pain: mouse models and specific blockers. *Pflugers Arch Eur J. Physiol.* **466**, 707–717 (2014). 24590509 10.1007/s00424-014-1484-4

[30] Isensee, J. et al. Depolarization induces nociceptor sensitization by CaV1.2-mediated PKA-II activation. *J. Cell. Biol.* **220**, e202002083 (2021). 34431981 10.1083/jcb.202002083 PMC8404467

[31] Clark, N. C. et al. Neurological phenotype and synaptic function in mice lacking the CaV1.3 α subunit of neuronal L-type voltage-dependent Ca2 + channels. *Neuroscience* **120**, 435–442 (2003). 12890513 10.1016/s0306-4522(03)00329-4

[32] Soons, P. A., Cohen, A. F. & Breimer, D. D. Comparative effects of felodipine, nitrendipine and nifedipine in healthy subjects: concentration-effect relationships of racemic drugs and enantiomers. *Eur. J. Clin. Pharmacol.* **44**, 113–120 (1993). 8453956 10.1007/BF00315467

[33] Marschallinger, J. et al. The L-type calcium channel Cav1.3 is required for proper hippocampal neurogenesis and cognitive functions. *Cell. Calcium*. **58**, 606–616 (2015). 26459417 10.1016/j.ceca.2015.09.007

[34] Zhang, H. et al. CaV1.2 and CaV1.3 neuronal L-type calcium channels: Differential targeting and signaling to pCREB. *Eur. J. Neurosci.* **23**, 2297–2310 (2006). 16706838 10.1111/j.1460-9568.2006.04734.x PMC3307544

[35] Sakamoto, K., Karelina, K. & Obrietan, K. CREB: a multifaceted regulator of neuronal plasticity and protection. *J. Neurochem*. **116**, 1–9 (2011). 21044077 10.1111/j.1471-4159.2010.07080.x PMC3575743

[36] Zhang, H. et al. Ca V 1.2 and Ca V 1.3 neuronal L-type calcium channels: differential targeting and signaling to pCREB. 10.1111/j.1460-9568.2006.04734.x 10.1111/j.1460-9568.2006.04734.x PMC3307544 16706838

[37] Altier, C. et al. The Cavβ subunit prevents RFP2-mediated ubiquitination and proteasomal degradation of L-type channels. *Nat. Neurosci.* **14**, 173–180 (2011). 21186355 10.1038/nn.2712

[38] di Biase, V. et al. Surface traffic of dendritic CaV1.2 calcium channels in hippocampal neurons. *J. Neurosci.* **31**, 13682–13694 (2011). 21940459 10.1523/JNEUROSCI.2300-11.2011 PMC3325119

[39] Boutin, M. E. et al. A multiparametric calcium signal screening platform using iPSC-derived cortical neural spheroids. *SLAS Discov.* **27**, 209–218 (2022). 35092840 10.1016/j.slasd.2022.01.003 PMC9177534

[40] Shmigol, A., Verkhratsky, A. & Isenberg, G. Calcium-induced calcium release in rat sensory neurons. *J. Physiol.* **489** (Pt 3), 627–636 (1995). 8788929 10.1113/jphysiol.1995.sp021078 PMC1156834

[41] Benham, C. D., Evans, M. L. & McBain, C. J. Ca2 + efflux mechanisms following depolarization evoked calcium transients in cultured rat sensory neurones. *J. Physiol.* **455**, 567–583 (1992). 1484362 10.1113/jphysiol.1992.sp019316 PMC1175659

[42] Zhang, D. et al. Single-nucleus transcriptomic atlas of glial cells in human dorsal root ganglia. *Anesthesiol. Perioper. Sci.* **1**, 17 (2023).

[43] Ambrosino, P., Soldovieri, M. V., Russo, C. & Taglialatela, M. Activation and desensitization of < scp>TRPV1 channels in sensory neurons by the PPARα agonist palmitoylethanolamide. *Br. J. Pharmacol.* **168**, 1430–1444 (2013). 23083124 10.1111/bph.12029 PMC3596648

[44] Naruse, K., McGehee, D. S. & Oxford, G. S. Differential responses of Ca-activated K channels to bradykinin in sensory neurons and F-11 cells. *Am. J. Physiol. Cell Physiol.* **262**, C453–C460 (1992). 10.1152/ajpcell.1992.262.2.C453 1539633

