# Supplementary material for: Voltage-gated calcium channels as key regulators of neuronal differentiation in the immortalized dorsal root ganglion neuronal cell line F11
Source: Sci Rep. 2026 Mar 23;16:14621. doi: 10.1038/s41598-026-44595-1 (PMC13153280; doi:10.1038/s41598-026-44595-1)
Supplement: Supplementary file 1 — Supplementary Material 1 [file 41598_2026_44595_MOESM1_ESM.docx]

**Voltage-gated calcium channels as key regulators of neuronal differentiation in the immortalized dorsal root ganglion neuronal cell line F11**

López D^1^, Brea J^1^, Barro M^1^, Loza MI^1*^, Martínez AL^1*^

^1^Innopharma Drug Screening and Pharmacogenomics Platform, BioFarma research group, Center for Research in Molecular Medicine and Chronic Diseases (CiMUS), Department of Pharmacology, Pharmacy and Pharmaceutical Technology, University of Santiago de Compostela and IDIS (Health Research Institute of Santiago de Compostela), Santiago de Compostela, Spain.

*Corresponding Author:

Email: [mabel.loza@usc.es](mailto:mabel.loza@usc.es) (MIL)

Email: [antonleandro.martinez@usc.es](mailto:antonleandro.martinez@usc.es) (ALM)

**Supplementary methods**

**Transfection efficiency assessment**

F11 cells were transfected with a GFP-encoding plasmid under the same experimental conditions used throughout the study (basal low-serum medium or differentiation medium). Plates were imaged on an Operetta CLS high-content imaging system (Revvity), and cell segmentation was performed in Harmony software using the Dynamic Phase Contrast (DPC) module to identify individual cells. GFP fluorescence was acquired using a 475 nm excitation/525 nm emission filter set. GFP-positive cells were defined by an intensity threshold set to the mean background fluorescence + 3 × S.D. (calculated from non-transfected control wells imaged in parallel under the same acquisition settings). Transfection efficiency was calculated per well as the percentage of GFP-positive cells relative to the total number of segmented cells.

**Microelectrode array (MEA) recordings**

Spontaneous spiking activity was recorded using a Maestro Pro system (Axion Biosystems, Atlanta, GA, USA). F11 cells were seeded onto CytoView 96-well MEA plates containing eight electrodes per well (M768-tMEA-96W, Axion Biosystems) at 7,500 cells/well and maintained under non-differentiation or differentiation conditions. After 72 h, plates were equilibrated for 15 min at 37 °C and 5% CO₂ in the recording chamber, and spontaneous spike activity was acquired for 15 s using AxIS Navigator (v3.9.1.1, Axion Biosystems). Spike detection was performed using a threshold set at 4× the standard deviation of baseline noise; all other acquisition and analysis parameters were kept at the AxIS default settings. Wells were included for analysis only if they met a quality criterion of eight active electrodes per well (as defined by AxIS). Mean firing rate (spikes/s) was computed per well using Neural Metrics Tool (v4.4.1, Axion Biosystems).

**Compartment analysis of VGCCs**

Compartment analysis of Ca_V_1.2/Ca_V_1.3 immunofluorescence was assessed in fixed, permeabilized and incubated F11 cells with a solution of primary antibodies as previously explained. 18 hours later, the cells were washed and incubated with a solution of goat anti-rabbit antibody conjugated with Alexa Fluor 647 (150083; Abcam), diluted 1:1000, Alexa 488 dye-conjugated anti-β-tubulin mouse antibody diluted 1:500, 1.5 µg/mL wheat germ agglutinin conjugated with Alexa Fluor 555 (W32464; Invitrogen, Paisley, UK) and 2.5 μg/ml Hoechst 33342 (H3570; Merck) in HBSS for 1 hour at room temperature. After washing twice with HBSS, a High Content Imaging System Operetta CLS was used to acquire bright field images and fluorescence signals (20x WD objective, 36 fields per well), using a 365 nm excitation/465 nm emission wavelength for Hoechst, 475 nm excitation/525 nm emission wavelength for Alexa 488, 525 nm excitation/575 nm emission wavelength for Alexa 555 and a 630 nm excitation/708 nm emission wavelength for Alexa 647. The WGA signal was used to define the cell boundary, Hoechst to segment nuclei, and β-tubulin to identify the neurite network; soma and neurite compartments were then defined within each segmented cell. Channel expression was quantified as Alexa Fluor 647 mean fluorescence intensity within soma and neurite regions.

**Supplementary figures**

**
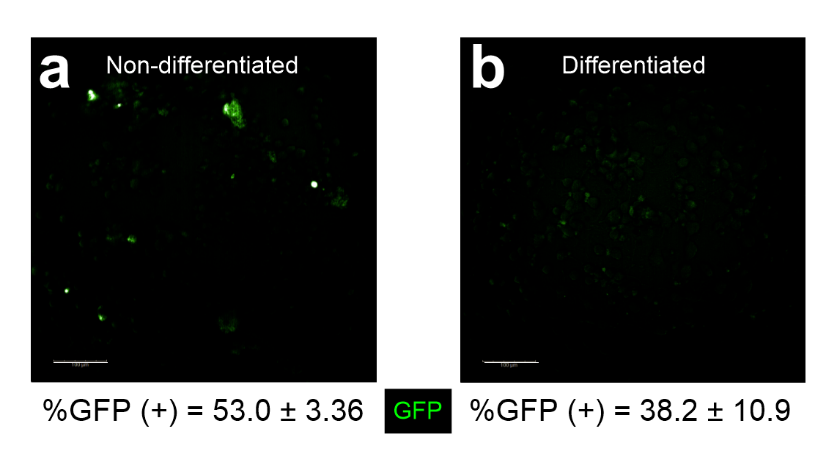
**

**S1 Fig. Transfection efficiency assessed using a GFP reporter construct. (a)** Representative image of F11 cells transfected with a GFP-encoding plasmid under basal (low-serum) conditions. (b) Representative image of F11 cells transfected with the same GFP construct under differentiation conditions. Transfection efficiency values shown under each image correspond to the percentage of GFP-positive cells per well (mean ± S.D.). Images and quantification correspond to three independent experiments (N=3) with two wells per condition (n=2).

**
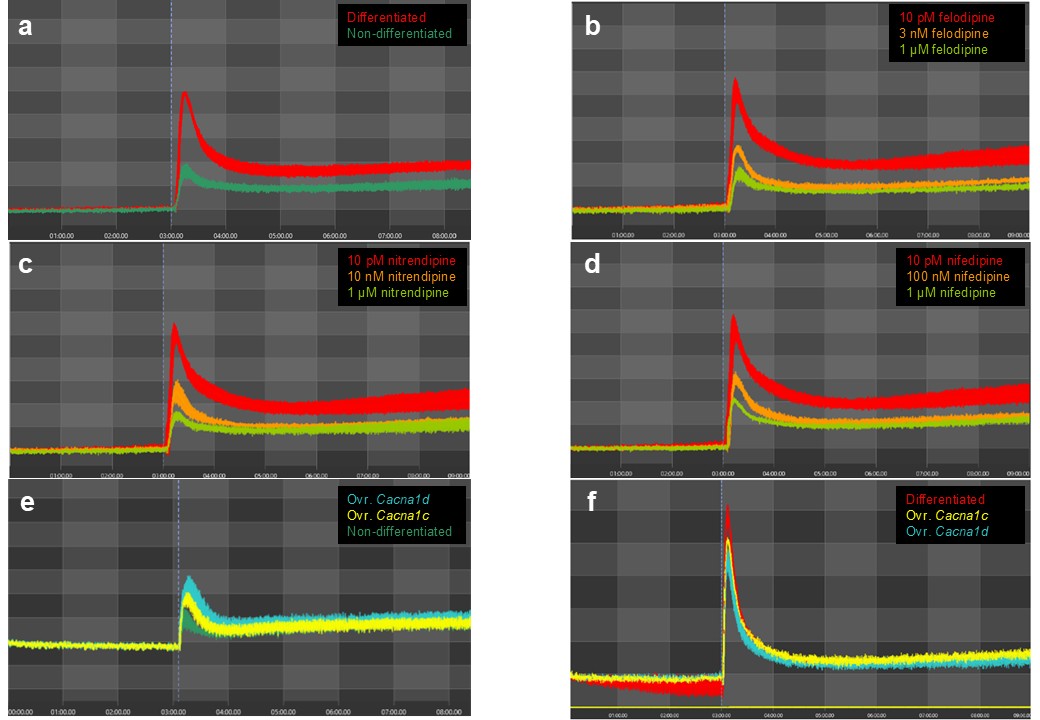
**

**S2 Fig**. **Representative well-averaged KCl-evoked intracellular Ca²⁺ traces across conditions. (a)** Overlay of non-differentiated and differentiated F11 cells. **(b)** Effect of felodipine at 10 pM, 3 nM (close to IC_50_) and 1 μM. **(c)** Effect of nitrendipine at 10 pM, 10 nM (close to IC_50_) and 1 μM. **(d)** Effect of nifedipine at 10 pM, 100 nM (close to IC_50_) and 1 μM. **(e)** Ca_V_1.2 and Ca_V_1.3 overexpression versus empty vector in non-differentiated F11 cells. **(f)** Ca_V_1.2 and Ca_V_1.3 overexpression versus empty vector under differentiation conditions. Traces were obtained under experimental conditions corresponding to those summarized in Figs. 1, 3, 6 and 7 and illustrate the population-level nature of the 30 mM KCl-evoked Ca²⁺ readout.

**
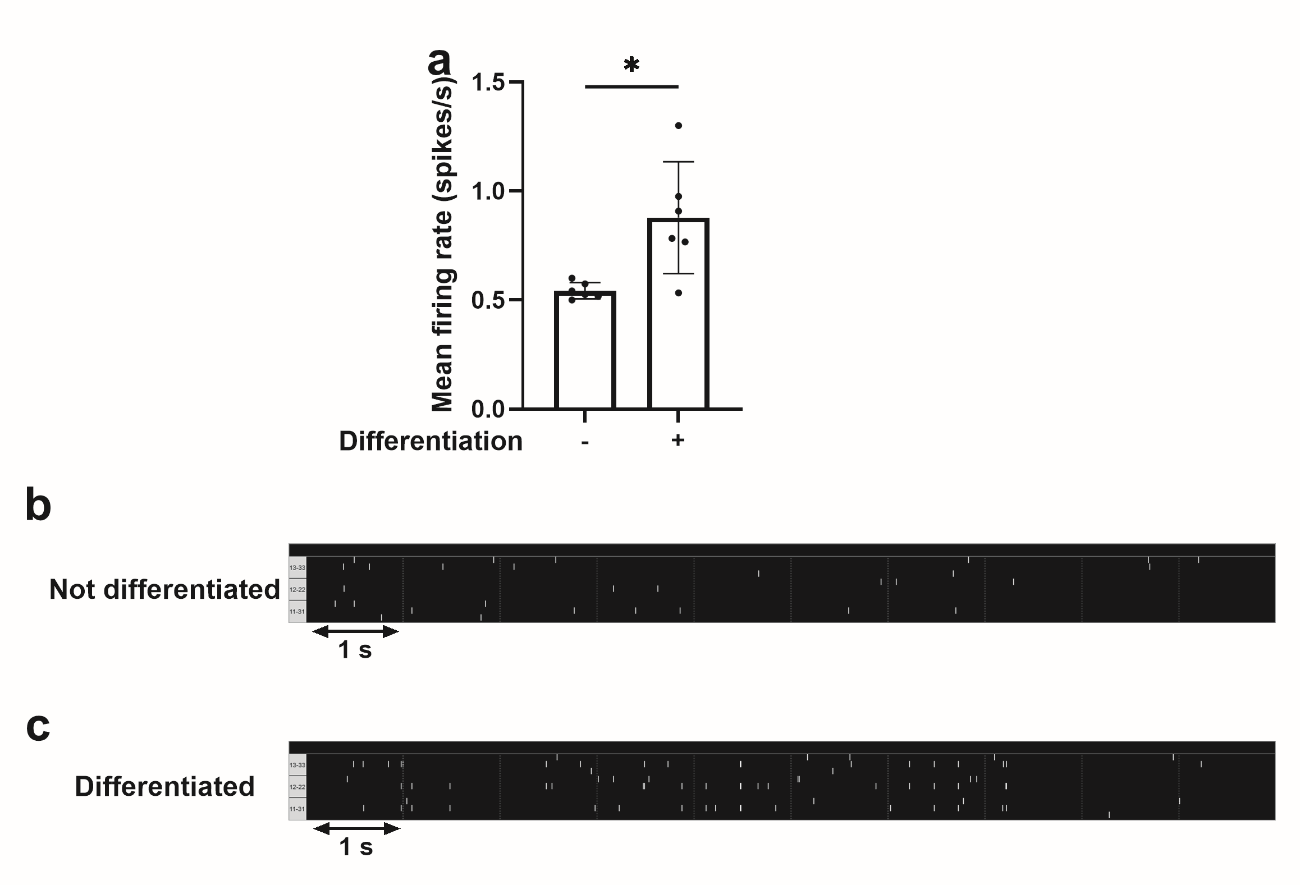
**

**S3 Fig**. **Differentiation increased spontaneous spiking activity in F11 cells measured by MEA.** **(a)** Mean firing rate (spikes/s) in non-differentiated (ND) and differentiated (D) F11 cells. Each data point represents one well (n=6 wells per condition), with 8 active electrodes per well. Spike detection threshold was set at 4×SD. *p < 0.05; Welch’s *t*-test. **(b)** Raster plot showing spike events over 10 s in a representative ND well across the eight electrodes. **(c)** Raster plot showing spike events over 10 s in a representative D well across the eight electrodes. Data are representative of one experiment out of four independent experiments (N=4).

**
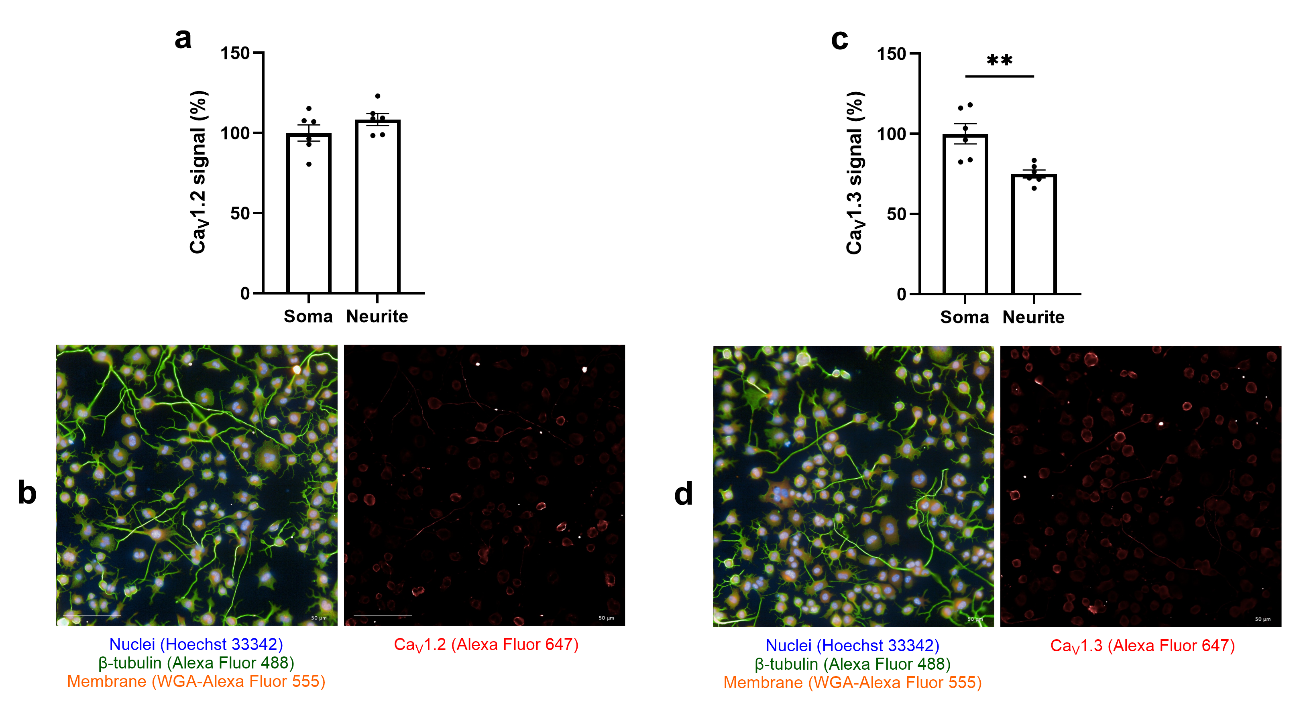
**

**S4 Fig. Harmony segmentation–based compartment analysis of Ca_V_1.2 and Ca_V_1.3 expression in soma versus neurites in differentiated F11 cells. (a)** Expression of Ca_V_1.2 VGCCs in soma and neurites of differentiated F11 cells (mean ± S.D.). **(b)** Representative images of differentiated F11 cells stained for nuclei (blue), cytoskeleton (green) and WGA (orange), for Harmony segmentation (left), and Ca_V_1.2 VGCCs (red) (right). **(c)** Expression of Ca_V_1.3 VGCCs in soma and neurites of differentiated F11 cells (mean ± S.D.). **(d)** Representative images of differentiated F11 cells stained for nuclei (blue), cytoskeleton (green) and WGA (orange), for Harmony segmentation (left), and Ca_V_1.3 VGCCs (red) (right). Each data point represents one replicate within the experiment (n = 4). Values in each graph are expressed as a percentage relative to the expression of Ca_V_1.2 or Ca_V_1.3 VGCCs, respectively, in the soma, which is defined as 100%. **p < 0.01; Welch’s *t*-test performed on non-normalized intensity values.

**
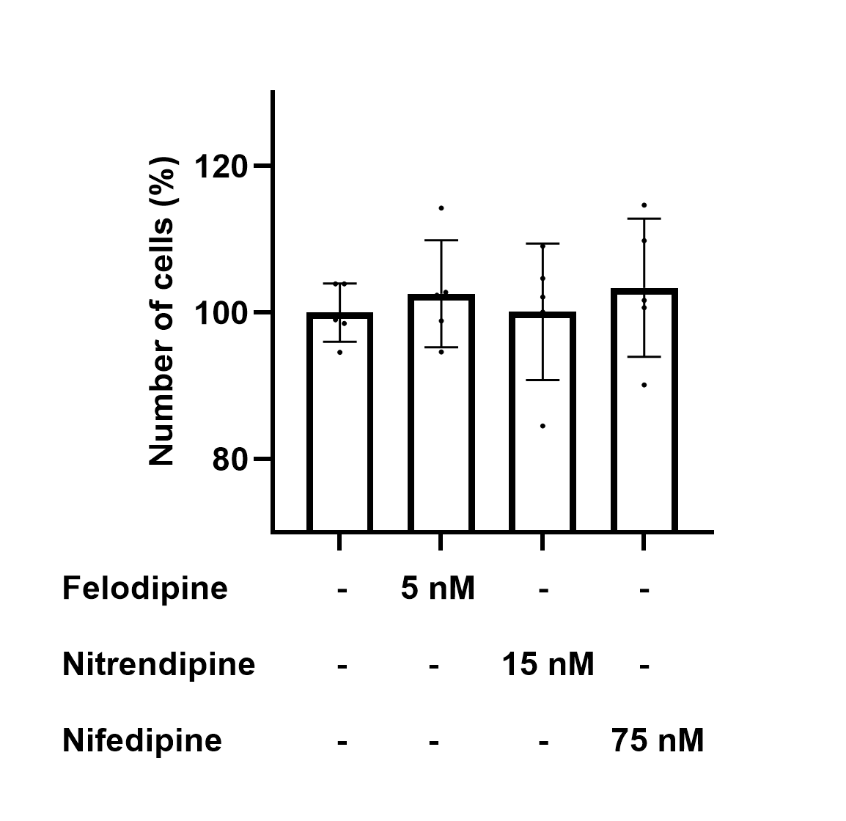
**

**S5 Fig**. **Exposure to 1,4-dihydropyridines during differentiation did not affect the viability of F11 cells.** Number of cells after exposure to 5 nM felodipine, 15 nM nitrendipine, and 75 nM nifedipine, compared to control differentiated F11 cells. Data are representative of one experiment out of three independent experiments (N=3), each with eight replicates per experiment (n=8). Values (mean ± S.D.) are expressed as a percentage relative to the number of control differentiated F11 cells not exposed to any 1,4-dihydropyridine, which is defined as 100%.

**S1 Table. RT–qPCR verification of Ca_V_1.2 and Ca_V_1.3 overexpression in F11 cells.** Data are presented from three independent experiments (N=3), each performed with (n=3) technical replicates per condition. ***p<0.001; Welch’s *t*-test.

| **Condition** | **Plasmid** | ***Target gene*** | **ΔC_q_ vs *36b4***  **(mean ± S.D.)** | **Statistical comparison**  **(significance)** |
| --- | --- | --- | --- | --- |
| Basal (low-serum) | Empty vector | *Cacna1c* | 9.663 ± 0.670 | <0.001*** |
| Basal (low-serum) | Ca_V_1.2 | *Cacna1c* | -0.545 ± 0.585 |  |
| Basal (low-serum) | Empty vector | *Cacna1d* | 18.7 ± 0.305 | <0.001*** |
| Basal (low-serum) | Ca_V_1.3 | *Cacna1d* | 0.631 ± 0.598 |  |
| Differentiation | Empty vector | *Cacna1c* | 6.300 ± 0.486 | <0.001*** |
| Differentiation | Ca_V_1.2 | *Cacna1c* | 0.017 ± 0.287 |  |
| Differentiation | Empty vector | *Cacna1d* | 17.9 ± 0.428 | <0.001*** |
| Differentiation | Ca_V_1.3 | *Cacna1d* | 0.752 ± 0.289 |  |

**S2 Table. Relative abundance of VGCC α1 subunit transcripts in non-differentiated (ND) and differentiated (D) F11 cells by RT–qPCR.**

| **Gene (symbol)** | **Channel type** | **ΔC_q_ vs *36b4* (ND)**  **(mean ± S.D.)** | **ΔC_q_ vs *36b4* (D)**  **(mean ± S.D.)** | **ΔΔC_q_**  **(D vs ND)** | **Fold change**  **2^-ΔΔCq^** |
| --- | --- | --- | --- | --- | --- |
| *Cacna1a* | Ca_V_2.1 | 10.628 ± 0.526 | 9.836 ± 0.308 | -0.792 | 1.73 |
| *Cacna1b* | Ca_V_2.2 | 6.860 ± 0.164 | 5.528 ± 0.224 | -1.332 | 2.52 |
| *Cacna1c* | Ca_V_1.2 | 9.124 ± 0.141 | 8.389 ± 0.265 | -0.736 | 1.67 |
| *Cacna1d* | Ca_V_1.3 | 20.770 ± 0.343 | 11.494 ± 0.123 | -9.276 | 620 |
| *Cacna1e* | Ca_V_2.3 | 13.345 ± 0.273 | 11.900 ± 0.327 | -1.445 | 2.72 |
| *Cacna1f* | Ca_V_1.4 | Not detected^†^ | Not detected^†^ | - | - |
| *Cacna1g* | Ca_V_3.1 | 8.925 ± 0.141 | 8.035 ± 0.173 | -0.891 | 1.85 |
| *Cacna1h* | Ca_V_3.2 | 7.443 ± 0.251 | 6.960 ± 0.160 | -0.483 | 1.40 |
| *Cacna1i* | Ca_V_3.3 | 10.298 ± 0.345 | 9.373 ± 0.269 | -0.925 | 1.90 |
| *Cacna1s* | Ca_V_1.1 | Not detected^†^ | Not detected^†^ | - | - |

^†^Not detected indicates no reliable amplification within the assay cycling conditions.

**S3 Table. Effect of overexpression (Ovr.) of Ca_V_1.2 and Ca_V_1.3 channels in F11 cells under basal and differentiation conditions on calcium response, maximum neurite length and intracellular ROS concentration compared to control F11 cells and statistical significance of the observed differences.** ***p<0.001, *p<0.05; ANOVA followed by Dunnett’s post-hoc analysis compared to control cells.

|  |  | **Basal conditions** | | | **Differentiation conditions** | | |
| --- | --- | --- | --- | --- | --- | --- | --- |
|  |  | Control cells | Ovr. Ca_V_1.2 (*Cacna1c*) | Ovr. Ca_V_1.3 (*Cacna1d*) | Control cells | Ovr. Ca_V_1.2 (*Cacna1c*) | Ovr. Ca_V_1.3 (*Cacna1d*) |
| **Calcium response (%)** | **Mean ± S.D.** | 100 ± 8.18 | 116.1 ± 9.55 | 123.8 ± 14.22 | 100 ± 11.15 | 91.33 ± 9.24 | 71.55 ± 15.57 |
|  | **p value** | - | 0.012* | <0.001*** | - | 0.160 | <0.001*** |
| **Maximum neurite length (%)** | **Mean ± S.D.** | 100 ± 6.61 | 110.4 ± 6.73 | 117.4 ± 17.54 | 100 ± 12.56 | 76.20 ± 6.46 | 47.51 ± 23.88 |
|  | **p value** | - | 0.236 | 0.035* | - | 0.040* | <0.001*** |
| **ROS concentration (%)** | **Mean ± S.D.** | 100 ± 6.71 | 105.6 ± 8.20 | 97.31 ± 11.88 | 100 ± 2.70 | 108.10 ± 3.32 | 113.9 ± 6.57 |
|  | **p value** | - | 0.480 | 0.833 | - | 0.011* | <0.001*** |
